# A Reactive Oxygen Species-Scavenging ‘Stealth’ Polymer, Poly(thioglycidyl glycerol), Outperforms Poly(ethylene glycol) in Protein Conjugates and Nanocarriers and Enhances Protein Stability to Environmental and Biological Stressors

**DOI:** 10.1021/jacs.2c09232

**Published:** 2022-11-11

**Authors:** Richard d’Arcy, Farah El Mohtadi, Nora Francini, Carlisle R. DeJulius, Hyunmoon Back, Arianna Gennari, Mike Geven, Maria Lopez-Cavestany, Zulfiye Yesim Turhan, Fang Yu, Jong Bong Lee, Michael R. King, Leonid Kagan, Craig L. Duvall, Nicola Tirelli

**Affiliations:** † Laboratory for Polymers and Biomaterials, Fondazione Istituto Italiano di Tecnologia, 16163 Genova, Italy; ‡ Division of Pharmacy and Optometry, School of Health Sciences, University of Manchester, Oxford Road, Manchester M13 9PT, U.K.; § Department of Biomedical Engineering, Vanderbilt University, Nashville, Tennessee 37235, United States; ∥ Department of Pharmaceutics, Ernest Mario School of Pharmacy, Rutgers, The State University of New Jersey, 160 Frelinghuysen Road, Piscataway, New Jersey 08854, United States; ⊥ Center of Excellence for Pharmaceutical Translational Research and Education, Ernest Mario School of Pharmacy, Rutgers, The State University of New Jersey, 160 Frelinghuysen Road, Piscataway, New Jersey 08854, United States

## Abstract

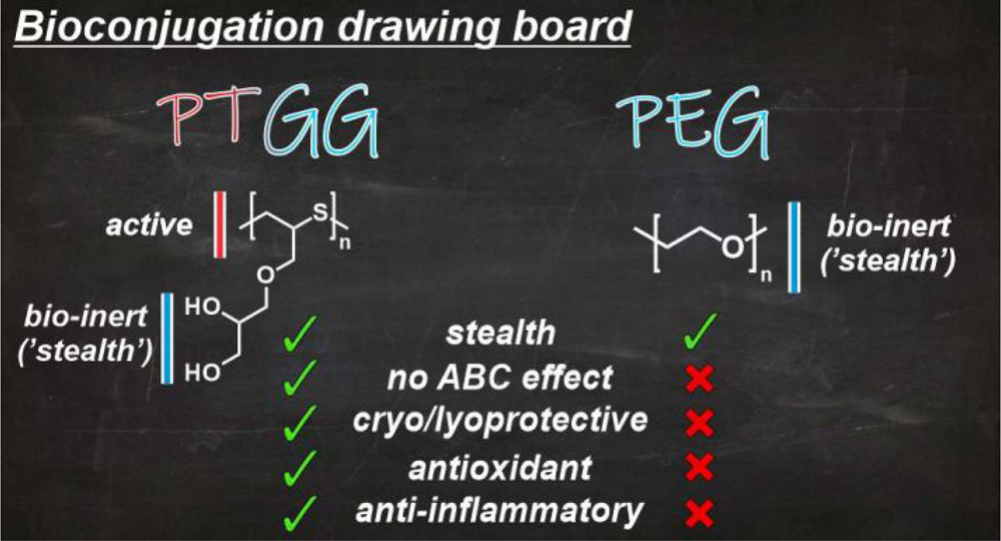

This study addresses well-known shortcomings of poly(ethylene glycol) (PEG)-based conjugates. PEGylation is by far the most common method employed to overcome immunogenicity and suboptimal pharmacokinetics of, for example, therapeutic proteins but has significant drawbacks. First, PEG offers no protection from denaturation during lyophilization, storage, or oxidation (e.g., by biological oxidants, reactive oxygen species); second, PEG’s inherent immunogenicity, leading to hypersensitivity and accelerated blood clearance (ABC), is a growing concern. We have here developed an ‘active-stealth’ polymer, poly(thioglycidyl glycerol)(PTGG), which in human plasma is less immunogenic than PEG (35% less complement activation) and features a reactive oxygen species-scavenging and anti-inflammatory action (∼50% less TNF-α in LPS-stimulated macrophages at only 0.1 mg/mL). PTGG was conjugated to proteins via a one-pot process; molar mass- and grafting density-matched PTGG-lysozyme conjugates were superior to their PEG analogues in terms of enzyme activity and stability against freeze-drying or oxidation; the latter is due to sacrificial oxidation of methionine-mimetic PTGG chains. Both in mice and rats, PTGG-ovalbumin displayed circulation half-lives up to twice as long as PEG-ovalbumin, but most importantly—and differently from PEG—without any associated ABC effect seen either in the time dependency of blood concentration, in the liver/splenic accumulation, or in antipolymer IgM/IgG titers. Furthermore, similar pharmacokinetic results were obtained with PTGGylated/PEGylated liposomal nanocarriers. PTGG’s ‘active-stealth’ character therefore makes it a highly promising alternative to PEG for conjugation to biologics or nanocarriers.

## Introduction

Over the past few decades, protein-based therapeutics have sharply changed the pharmaceutical landscape, with the provision of life-critical drugs such as insulin and liraglutide (Victoza) and blockbusters such as adalimumab (Humira). However, their use is often hampered by unsatisfactory physico-chemical stability (against factors such as oxidation, lyophilization, temperature, pH), immunogenicity, and pharmacokinetics. Conjugation to “stealth” polymers, and in particular to poly(ethylene glycol) (PEG), can remarkably enhance plasma half-life and biodistribution, leading to numerous PEGylated therapeutics reaching the market. Proteins represent the largest subgroup of approved PEGylated therapeutics (currently at 26), with a market value estimated to have been $7.7 billion worldwide back in 2017.^1^

Unfortunately, concerns over PEG’s own immunogenicity have grown, although this appears to be drug- and patient- specific, with a genetic predisposition now established.^2^ For example, PEGylated asaparginase (Oncaspar), which has a half-life of 5.7 days (1.3 for unmodified enzyme),^3^ is associated with a significant incidence of PEG-immunogenicity (∼25% of patients^4^), with complement activation believed to be the critical modulator of this phenomenon.^5^ The downstream immunogenic effects are known as hypersensitivity reactions (HSRs, or infusion reactions) and accelerated blood clearance (ABC), that is, the more rapid clearance from blood plasma upon repeated dosing. Although originally considered disparate immunogenicities, seminal work by Kozma et al.^6^ has found that these reactions are actually ‘two sides of the same coin,’ sharing common initiation events, for example, anti-PEG IgM or IgG opsonization followed by classical complement activation. These immunogenicity issues have halted the clinical evaluation of pegnivacogin (Revolixys)^7^ and resulted in the market withdrawal of peginesatide (Omontys)^8^ and pegloticase (Krystexxa).^6^ They are also known to occur in various other clinically approved PEGylated-formulations such as mono-mPEG-epoetin-β (Mircera),^9^ pegvaliase-pqpz (Palynziq),^10^ and Doxil.^11^ Matters are further complicated by the presence of “pre-existing” anti-PEG antibodies in the treatment-naivë population, which in the last two decades have increased from 0.2% to between 44^12^ and 72%^13^ of the population, arising due to the use of PEG in cleaning products and foods.^13^ Furthermore, the widespread use of PEG in Covid-19 vaccines and boosters has led to significantly higher anti-PEG levels found in those vaccinated, further bringing in to question the future of PEGylated therapeutics.^14^

Therapies based on PEGylated actives have also been linked to intracellular vacuolation in a variety of organs such as duodenum, heart, kidneys, liver, and spleen because of the long-term persistence of intact PEG in the body.^15^ In short, (bio)degradable and nonimmunogenic alternatives to PEG are urgently sought. Poly(2-methyl-2-oxazoline) (PMOX) recently emerged as a potential PEG alternative owing to its impressive blood circulation times and ‘ultrahigh’ drug loading it permits to micellar drug-carriers.^16^ However, concern remains over its propensity to activate complement/immunogenicity;^17^ for example, similarly to PEG, PMOX presents ABC upon repeated dosing.^18^ Other potential alternatives to PEG are poly(dimethylacrylamide) (PDMA), poly(hydroxypropyl methacrylamide) (PHPMA), and poly(vinylpyrrolidone) (PVP), which have less impressive circulation half-lives but nevertheless perform better than PEG and PMOX in multiple dosing regimes, with an apparent lack of ABC.^18^ Trimethylamine *N*-oxide-derived and carboxybetaine polyzwitterions have also emerged as highly promising PEG alternatives as both provide improved plasma circulation times in mice (up to 3.6-fold increased *t*_1/2β_ vs PEG)^19^ with no discernible ABC effect.^19,20^ More recently, polysulfoxides have demonstrated an appealingly low complement activation and increased plasma circulation (2.7-fold increase in first-order terminal elimination constant) with respect to PEG.^17b,21^ Interestingly, polysulfoxides open a new paradigm of ‘active-stealth’ because they also display antioxidant and thus also anti-inflammatory properties.^17b^ Polysulfoxides are also degradable: although synthesized via controlled oxidation of polysulfides, further oxidation to sulfones leads to chain fragmentation,^22^ which is appealing to reduce the risk of long-term accumulation.

Evolving this concept of ‘active-stealth,’ we have taken inspiration from α2-macroglobulin, an important proteinase inhibitor that functions in highly inflammatory, reactive oxygen species (ROS)-rich environments. While originally thought to be resistant to oxidative denaturation, it was later found that its stability is due to surface-exposed methionines, acting as sacrificial substrates, that protect an activity-critical tryptophan from oxidation.^23^ This sacrificial protection may hold general validity, because ex or in vivo oxidation of therapeutic proteins is well known to be deleterious for both their shelf-life and efficacy, with the ensuing denaturation and/or aggregation phenomena potentially leading to shorter half-lives and higher immunogenicity.^24^ For example, efforts to exploit galectin-1, a potent immunomodulator, have been hampered by its ROS-sensitivity leading to aggregation and loss of activity in inflammatory environments.^25^ However, it is important to note that oxidation can occur at any stage of a therapeutic protein’s lifecycle, from synthesis, purification, and storage, to biological setting.^24b^

**Scheme 1:**
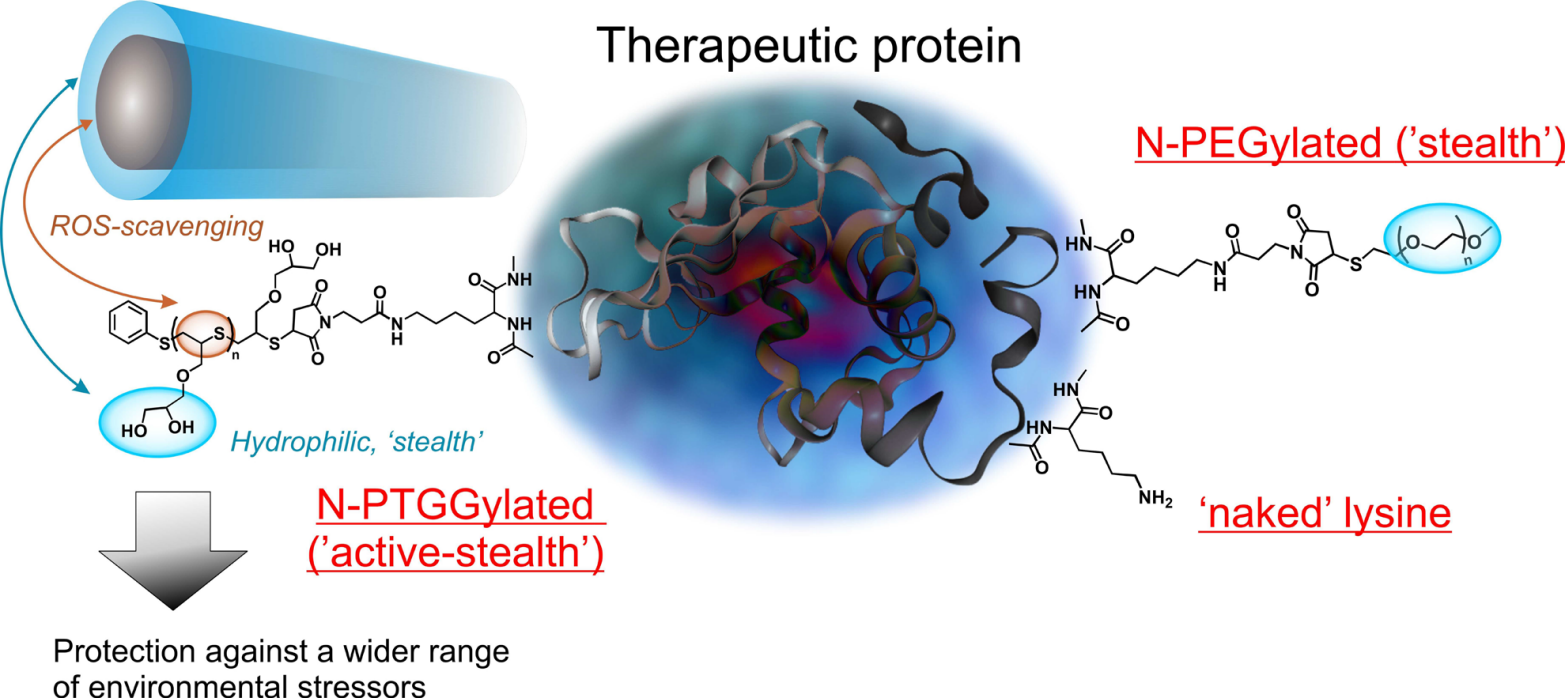
PTGG (Left) Thioethers Allow for Protection against Oxidants (ROS), while Its Hydrophilic Glycerols Provide a ‘Stealth’ Behavior (Lower Immunogenicity, Higher Stability against Degradation, and Denaturation) Similar to or Better Than PEG (Right)

With an analogy to α2-macroglobulin, we have developed a hydrophilic methionine-mimetic prosthetic to graft to proteins of interest: poly(thioglycidyl glycerol) (PTGG) (Scheme 1). As a polysulfide, PTGG is a potent ROS scavenger^26^ (hence also an anti-inflammatory agent^27^), while its glycerol side chains offer (i) a stealth nature; glycerol-containing macromolecules are shown to have remarkably low fouling,^28^ have often outperformed PEG as stealth polymers (1.5^29^–2-fold^30^ longer *t*_1/2β_), and do not display ABC^31^ and, (ii) like glycerol,^32^ have cryo/lyoprotective properties.^33^ In this regard, polyols are typically more protective than analogous low MW alcohols.^34^ In a pioneering example, Maynard and co-workers showed that a polymeric trehalose provided better cryo/lyoprotection than the parent disaccharide and even more so when directly conjugated to, for example, lysozyme.^35^ Endowing proteins of both cryo/lyo- and ROS- protection would therefore be advantageous to prevent oxidative damage both in the body and during manufacturing, for example, from vapor phase H_2_O_2_ contamination used for sterilization,^36^ during the freeze-drying process,^37^ or during storage (Fenton metal contaminant/UV/O_2_ catalyzed (photo-)oxidation).^24b,37b,38^

As models, we have used (A) lysozyme for in vitro tests, due to its sensitivity to, for example, lyophilisation^35^ and oxidation, and in vivo both (B) ovalbumin (OVA), whose antigenicity is known to stimulate anti-PEG antibodies and ABC,^39^ and (C) liposomes, a classical nanocarrier prone to ABC.

## Results and Discussion

### Synthesis of Glycol Polysulfides

**Figure 1:**
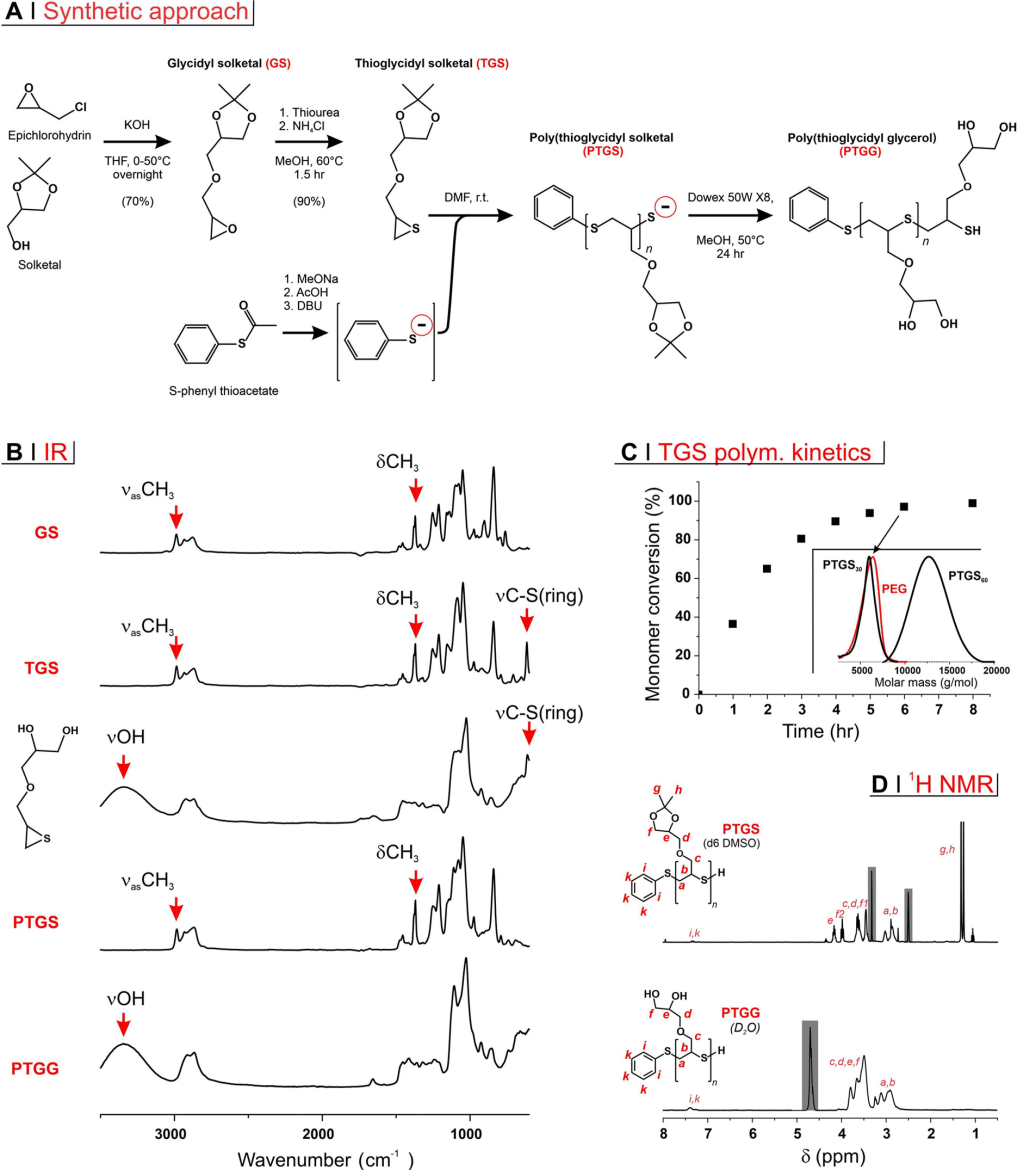
(A) TGS was produced via a two-step reaction sequence and then used in the episulfide ROP initiated by an in situ formed phenyl thiolate; the resulting PTGS was treated with an acidic resin, to deprotect glycerol side chains and protonate its terminal thiol thereby producing the final PTGG. (B) IR spectra of compounds prepared during PTGG synthesis; episulfide rings can be monitored through the typical C–S stretching vibration of three-membered rings located at ∼600 cm^–1^. Glycol deprotection (both in the low molecular weight TGS and in the polymeric PTGS) corresponds to the loss of the methyl stretching and bending vibrations of the isopropylidene group and to the appearance of an OH stretching vibration. (C) TGS consumption kinetics as determined by ^1^H NMR in deuterated-DMF (reduction in intensity in the resonance at 2.90–2.92 ppm of episulfide ring protons [−CH_2_S−]CH−); theoretical DP = 30). In the inset, GPC traces (RI signal, triple detection in THF); the sample collected after 6 h of polymerization (PTGS30) is highlighted with an arrow; the other two refer to a 12 h of polymerization with a theoretical degree of polymerization = 60 (PTGS60) and to mPEG thioacetate; the latter and PTGS30 have very similar molecular weight distributions. (D) ^1^H NMR spectra of PTGS30 in deuterated DMSO and PTGG30 in D_2_O; letters correspond to the assignments on the chemical structures and shadowed areas to solvent peaks.

The functional monomer at the basis of this study was prepared in a two-step reaction sequence (Figure 1A), where first the epoxide/protected diol glycidyl solketal (GS) is produced via etherification of epichlorohydrin with solketal; the epoxide group of GS was then converted into an episulfide via reaction with thiourea, yielding thioglycidyl solketal (TGS) and urea as a byproduct. All these reactions can be easily followed via IR spectroscopy, because of the presence of a number of diagnostic bands (Figure 1B). TGS was polymerized via anionic ring-opening polymerization (ROP) using an in situ generated initiator (Figure 1A, bottom).

We have previously proven the advantage of using in situ generated thiolates from thioacetates; when used in combination with a phosphine as an internal reducing agent, this procedure avoids the occurrence of disulfides, which act as chain transfer agents in the episulfide ROP.^40^ Importantly, thiolates are produced with a large organic counterion (DBU), because such ‘naked’ thiolates propagate quicker than those of smaller inorganic cations.^41^ Indeed, the ROP kinetics proceeded fast with virtually complete monomer consumption after 6–8 h with a monomer/initiator molar ratio = 30 (Figure 1C). 6 h was therefore used as a polymerization time for the synthesis of PTGS30, while 12 h was employed for a polymer with twice the theoretical degree of polymerization, PTGS60. The two polymers showed actual degrees of polymerization close to their theoretical values and very narrow molecular weight distributions (Table 1). Of note, PTGS30 is very similar in size to the commercially available 5 kDa PEG monomethyl ether (mPEG-OH); this prompted us to synthesize a (protected)thiol-bearing PEG as a size- and reactivity-matched control; mPEG-SAc (bearing a terminal thioacetate) was prepared from mPEG-OH via a facile, one-step Mitsunobu reaction recently developed by two of the authors,^42^ which can be readily (and also in situ) converted to a thiol via treatment with sodium borohydride.

**Table 1:** Molecular Mass Data of the Polymers Used for Bioconjugation Reactions

	*M*_n_® (g/mol)/DP_n_®			*Đ*^b^
	theor.	^1^H NMR^a^	GPC^b^	
PTGS30	6230/30	6026/29	5470/26.3	1.08
PTGS60	12,350/60	11,330/55	12,250/59.5	1.06
mPEG-OH^c^	5030		5390	1.08
mPEG-SAc^c^	5080		5320	1.09

a ^1^H NMR was used to obtain the number average degree of polymerization DP_n_® (ratio between the of the solketal methyl resonances at 1.26 and 1.31 ppm and that of the phenyl initiator at 7.21 ppm), then calculating the corresponding *M*_n_®.

b Triple detection GPC in THF. A small amount of tributylphosphine was added prior to injection to ensure terminal thiols.

c mPEG-SAc was derived from its parent polymer, mPEG-OH and in turn used to generate in situ mPEG-SH. The data here reported ensure that the PEG chain is comparable in dimensions to PTGS30, and this has not been altered by its functionalization.

Using an acidic ion exchange resin (Dowex 50WX8), PTGS30 and PTGS60 were finally deprotected to the corresponding glycerol-bearing and thiol-terminated PTGG. The quantitative character of the deprotection was confirmed by ^1^H NMR spectrometry (disappearance of methyl resonances in Figure 1D).

### PTGG vs PEG: Biocompatibility

Biocompatibility of a material can be accurately defined only by detailing its specific application. Having in mind the use of PTGG as a potential alternative to PEG for the purpose of bioconjugation to therapeutic proteins, a biocompatibility assessment ought to evaluate any direct cytotoxic effects, as well as potential for recognition as a foreign body. We have assessed these points in vitro through assays of cytotoxicity, uptake in phagocytic cells, and complement activation (alternative pathway).

**Figure 2:**
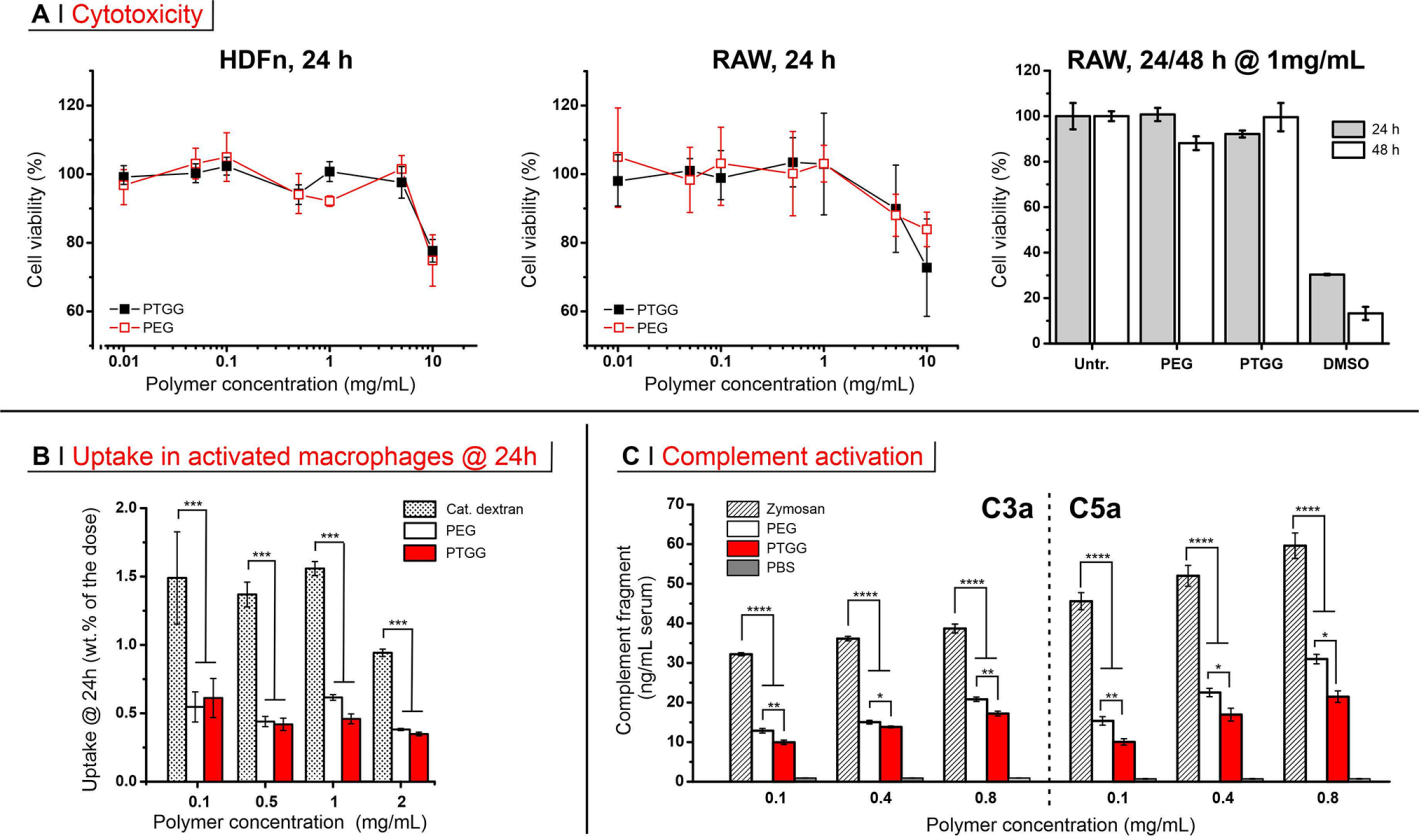
(A) Viability (mitochondrial activity via MTS assay) of HDFn (left) and RAW 246.7 macrophages (center) after 24 h of incubation as a function of the concentration of PTGG30 or mPEG (5 kDa) for 24 h (*n* = 3); viability of RAW cells was also assessed after treatment with PTGG and PEG at concentration of 1 mg/mL for 24 or 48 h. Cells were also treated with 5% DMSO as positive control. (B) Uptake of FITC-labeled PTGG30 and mPEG into RAW 246.7 macrophages after 24 exposure (fluorescence measured in cell lysates, *n* = 3). (C) Complement activation by PTGG30 or mPEG as assessed through the production of two soluble markers, C3a (left) and C5a (right), in human serum. Zymosan and PBS were used, respectively, as the positive and negative control (*n* = 3). Statistical significance: one-way analysis of variance (ANOVA) with a Tukey’s means comparison; *P ≤ 0.05, **P ≤ 0.01, ***P ≤ 0.001, and ****P ≤ 0.0001.

At 24 h, both a nonphagocytic cell model (human nenonatal dermal fibroblasts, HDFn) and a phagocytic model (murine RAW 246.7 macrophages) showed almost identical toxicity profiles for PTGG and the size-matched (5 kDa) PEG (Figure 2A, left and center), both virtually having no effect on a cell viability up to a concentration of 5 mg/mL. Because of their phagocytic nature, RAW cells can be a more sensitive toxicity indicator; yet, even at 1 mg/mL for 48 h, their viability was not significantly decreased (Figure 2A, right). The two polymers behaved very similarly also in terms of cellular uptake, which was quantified through the presence of fluorescently labeled PTGG30 and PEG in the cell lysates of preactivated (500 ng/mL lipopolysaccharides, LPS) RAW upon 24 h incubation (Figure 2B; see also Supporting Information, Figure S8). Through the 0.1–2 mg/mL concentration range, the amount of internalized material was considerably lower than that of a positive control (cationic dextran); most importantly, the fraction of internalized dose was essentially constant for both polymers. This suggests that their internalization is predominantly a (macropinocytosis-based) unspecific uptake of the liquid phase, that is, the absence of specific recognition hence a good potential as stealth polymers. Finally, PTGG appeared to produce a lower (alternative) complement activation (assessed by the generation of C3a and C5a anaphylatoxins, Figure 2C) than PEG, and considerably lower than zymosan, used as a positive control: at a concentration of 0.1 mg/mL, PTGG produced 60 or 23% less C3a, and 66 or 35% less C5a than respectively zymosan and PEG. The alternative complement activation is typically based on the cleavage of a thioester in the C3b fragment of the C3 protein by nucleophiles such as alcohols. Many polyols such as poly(2-hydroxyethylmethacrylate) (PHEMA)^43^ and cellulose^44^ are indeed known complement activators, although this behavior is predominantly ascribed to their primary alcohols, such as those in the 6-position of sugars that are up to 7 times^45^ more C3b-reactive than secondary alcohols. Interestingly, the combination of a primary with a vicinal secondary alcohol (1,2-diols/glycols), as in PTGG side chains, shows a peculiarly low complement activation; for example, linear and branched polyglycerols show a low complement activation,^46^ significantly lower than zymosan (possessing a strongly activating 6-position primary alcohol and no 1,2-diols), but also lower than PEG,^30^ which tallies with their comparatively longer circulation.^29,30^ We are inclined to ascribe this favorable behavior of glycols to the possibility of internal hydrogen bonding between the two alcohols, which decreases their nucleophilicity; this is best exemplified by 2-deoxyglucose which has a 2-fold higher C3b reactivity than glucose (despite the same structure with one alcohol group less).^45^

### PTGG vs PEG: ROS Scavenging Ability

**Figure 3:**
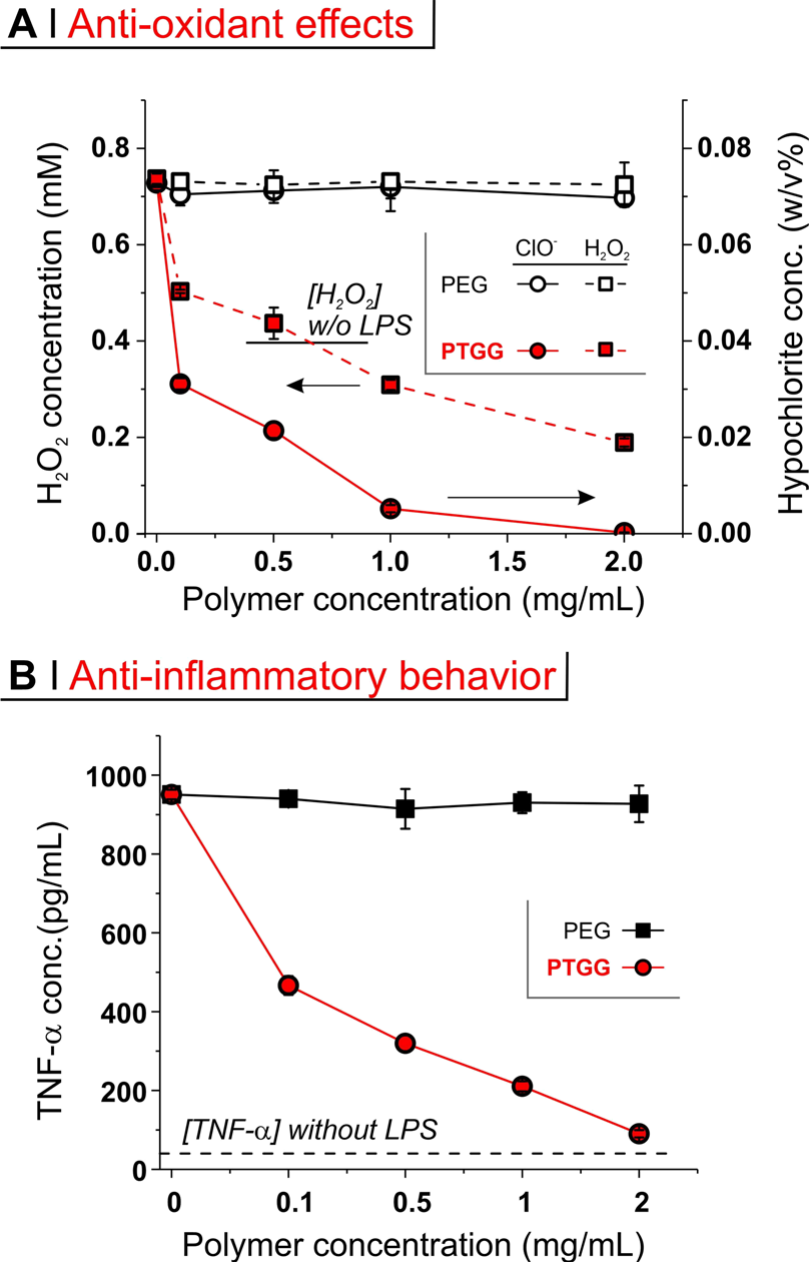
(A) Hydrogen peroxide (left axis, squares) and hypochlorite (right axis, circles) concentration in the cell lysates of RAW macrophages preactivated with 500 ng/mL LPS and exposed for 24 h to mPEG or PTGG30. Without LPS stimulation, hypochlorite was not detected, while H_2_O_2_ was present at about 0.4 mM, and it is noteworthy that high concentrations of PTGG reduced [H_2_O_2_] considerably below this basal level. (B) TNF-α concentration in RAW supernatants of preactivated RAW macrophages exposed to mPEG or PTGG30 for 24 h.

In this study, we focused on two of the most representative members of the ROS family: hydrogen peroxide and hypochlorite. Upon exposure to LPS activation, RAW upregulates the production of both ROS, and the presence of PEG did not affect their levels (black symbols in Figure 3A). Conversely, PTGG triggered a potent reduction in both, particularly hypochlorite: the cellular levels of H_2_O_2_ and hypochlorite were respectively reduced by ∼75% (already below the levels of nonstimulated cells) and >90% at a PTGG concentration of 1 mg/mL.

In parallel to ROS scavenging, PTGG caused a remarkable dose-dependent reduction in TNF-α levels (∼50% reduction at only 0.1 mg/mL and ∼90% at 2 mg/mL). This is coherent with our previous work on antioxidant polysulfide nanoparticles which displayed potent antioxidant and anti-inflammatory effects on primary glial cells in vitro, while in vivo were able to significantly reduce the severity ischemic stroke in a murine model.^27c^ Given the critical role of ROS in the toxicity and immunogenicity of nanomedicines,^47^ PTGG conjugates may have the ability to mitigate these effects, as well as act synergistically in therapies targeted toward diseases with strong inflammatory characters, as has been recently demonstrated with drug-loaded polysulfide micellar systems.^48^ We do however caveat this anti-inflammatory behavior described here in Figure 3 to be specifically counteracting an innate immune response and not to be confused with an antiadaptive/humoral immune response.

### PTGG Conjugation to a Model Protein: Lysozyme

#### (A) Synthesis and Characterization of PTGG vs PEG Lysozyme Conjugates

Lysozyme is a long-time standard model for studies of protein/enzyme conjugation to a variety of pre-formed synthetic polymers, using, for example, cysteines for Michael-type addition on maleimide-terminated polymers,^49^ or lysines for reductive amination on terminal aldehydes^50^ or active esters^51^/carbonates.^21a^

**Scheme 2:**
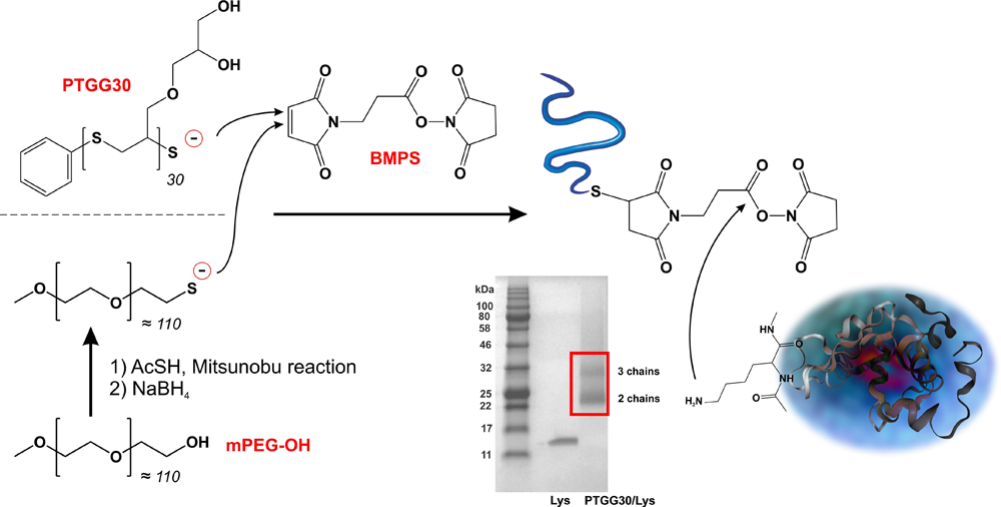
Conjugation of Thiol-Terminated PTGG and PEG to Lysozyme via the Heterobifunctional Linker β-Maleimidopropionic Acid *N*-Hydroxysuccinimide Ester (BMPS)

Here, for a covalent conjugation of PTGG chains to lysozyme, we have adopted a variation to the latter (lysine-reactive) approach: a two-step, one-pot procedure based on a heterobifunctional linker (BMPS) which is first reacted with the PTGG terminal thiol at its maleimide end and then with lysozyme lysines at its active *N*-hydroxysuccinimide ester (Scheme 2; please note that SDS-PAGE refers to PTGG30/Lys). Of note, PTGG was first treated with sodium borohydride to reduce any disulfide, before reacting in a 1:1 molar ratio with BMPS, and then with lysozyme, typically at a 5:1 active ester/lysine molar ratio.

The number of polymeric chains per lysozyme was qualitatively assessed via gel electrophoresis (SDS PAGE) and quantitatively confirmed through the protein weight fraction of the conjugates (bicinchoninic acid (BCA) assay), as shown in Table 2. Using a 5:1 thiol/lysine molar ratio, an average of about 2.5 chains of PTGG were grafted on lysozyme, which corresponds to having just under half the free amines reacted; the procedure did not appear to be affected by PTGG molecular weight (same number of grafted chains for PTGG30 and PTGG60). mPEG was grafted with higher efficiency: the conjugate mPEG/Lys[2] was synthesized using the same thiol/lysine molar ratio as PTGG-conjugates but featured an almost twice larger number of chains per lysozyme; to obtain a comparable derivatization, the thiol/lysine ratio had to be lowered to 2.5 (mPEG/Lys[1]).

**Table 2:** Characterization of Lysozyme and of Its PTGG and mPEG Conjugates

	SH/lysine ratio^a^	chains/lysoz.^b^		enzymatic activity	
		SDS PAGE	protein cont.	10^3^ × *k* (min^–1^)^c^	norm. A2 (%)^c^^,^^d^
lysozyme	0	0	0	76 ± 5	100 ± 0.8
PTGG30/Lys	5	2–3	2.5 ± 0.2	74 ± 13	80.6 ± 2.8
PTGG60/Lys	5	2–3	2.7 ± 0.4	18 ± 3	106.6 ± 10.5
mPEG/Lys[1]	2.5	2–3	2.9 ± 0.1	23 ± 3	91.0 ± 7.2
mPEG/Lys[2]	5	4–5	4.3 ± 0.3	5 ± 7	87.3 ± 4.2

a In the conjugation reaction.

b Evaluated qualitatively from the size of the most intense bands in gel electrophoresis (SDS-PAGE) and more quantitatively from the protein content per gram of material (BCA assay, *n* = 3).

c The fluorescence of the substrate (EnzChek Lysozyme Assay Kit) increased with time, and it was fitted with an exponential growth equation (fluorescence = A1 × exp(time/τ) + A2); the rate constant is expressed as *k* = 1/τ, the fluorescence at plateau is A2.

d The fluorescence at plateau (A2) was normalized as a % of the unconjugated lysozyme.

The reactivity of maleimides with thiols is extremely high,^52^ making it unlikely for them to discriminate between a primary thiol (PEG) and a secondary one (PTGG). We are inclined to ascribe PTGG ‘s lower—but independent of molecular weight—grafting to the local steric hindrance of glycerol side chains onto the PTGG-BMPS adduct active ester.

#### (B) Enzymatic Activity

**Figure 4:**
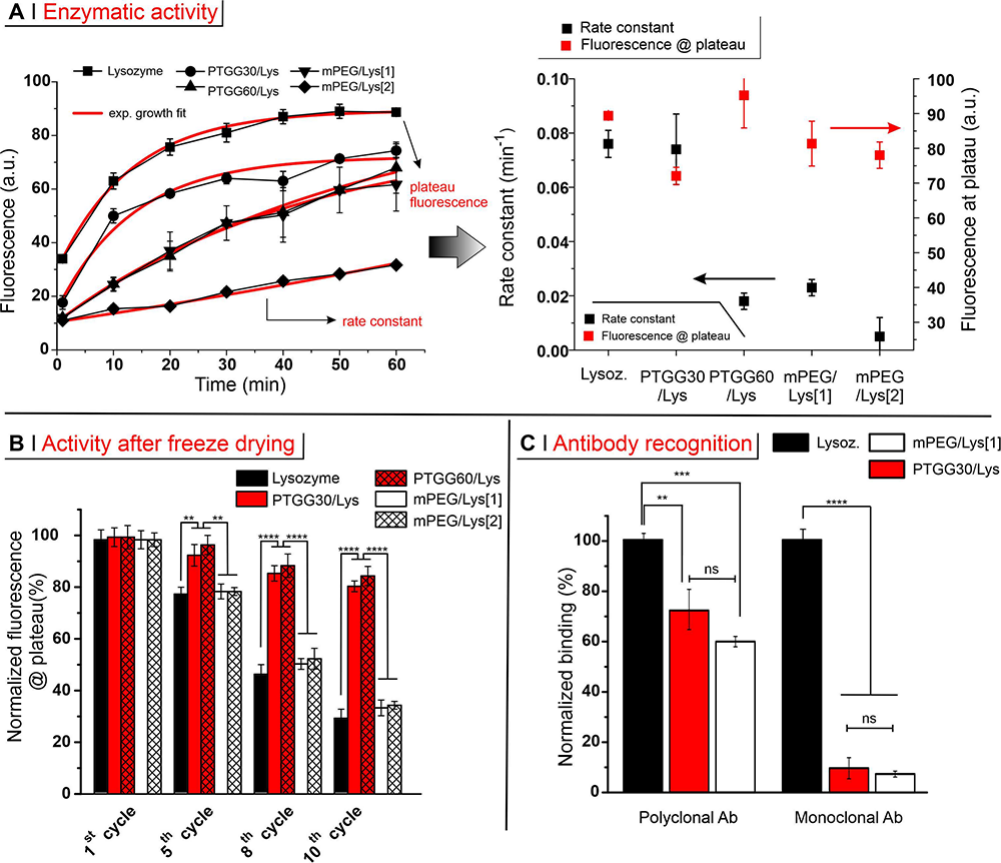
(A) Lysozyme activity was assessed using a dye-quenched assay, keeping the protein concentration constant at 0.5 mg/mL; the fluorescence was monitored over 60 min at 37 °C, fitting the data with an exponential growth equation fluorescence = A1 × exp(time/τ) + A2 (inset; red lines are fittings). A rate constant is calculated as 1/τ, while the sum A1 + A2 provides the fluorescence at plateau. (B) Relative activity of lysozyme and of its conjugates after 1, 5, 8, and 10 lyophilization cycles. For both systems, the activity before freeze drying is considered 100%. (C) Binding of monoclonal or polyclonal antibodies to lysozyme derivatives was measured by direct enzyme-linked immunosorbent assay (ELISA). Because free lysozyme is adsorbed on the plate surfaces considerably more than its conjugates, the readings were first normalized against the amount of adsorbed enzyme adsorbed (quantifying the residual lysozyme in solution via a BCA assay), effectively providing an amount of antibody per amount of adsorbed (conjugated) protein. The results were then normalized by considering free lysozyme as 100%. Statistical significance: one way ANOVA with a Tukey’s means comparison; *P ≤ 0.05, **P ≤ 0.01, ***P ≤ 0.001, ****P ≤ 0.0001.

The conjugation of polymer chains to an enzyme can significantly decrease its activity; for example, commercially available PEG-asparaginase has about half of the activity of the parent enzyme.^53^ Here, we have assessed lysozyme activity through an assay based on a broad mixture of dye-quenched substrates (*Micrococcus lysodeikticus* cell wall lysates); fluorescence therefore increases with the extent of substrate degradation. By fitting the corresponding kinetics with an exponential growth model (inset in Figure 4A; fittings are shown as red lines), one obtains two parameters that account for different effects on the enzyme activity:

(1) the fluorescence at plateau; its reduction would correspond to a lower breadth of substrates processable by the active site, thereby specifically accounting for modifications at or around the active site.

(2) A rate constant; its reduction would reflect a lower number of active enzymes and/or their lower turnover rate, which may be caused by events occurring also distant from their active site; for example, whole protein denaturation or a more difficult approach of substrates to the active site because of steric hindrance.

As shown in Figure 4A, the fluorescence at plateau (red symbols) was relatively constant for all derivatives, suggesting that all conjugates have the same breadth of activity as the parent lysozyme, and that the active site survived the bioconjugation relatively untouched. The rate constant was, however, significantly lowered by both high and low degrees of PEGylation and by the presence of PTGG60 chains, but only marginally by that of the shorter PTGG30. It is worth noting that the relatively bulky glycerol side chains likely endow PTGG with a larger persistence length (i.e., the macromolecule is considerably less coiled and appears ‘more straight’) than PEG, that is, for a similar size, PTGG is likely less coiled.

Assuming the pattern of conjugation to be similar for all conjugates, PTGG30 would thus be expected to cause less steric hindrance than both mPEG (because less coiled) and PTGG60 (because shorter), which tallies with the effects on the rate constant. We therefore assume that bioconjugation has not significantly denatured the protein or affected the active site, but the access to the active site remains substantially unrestricted only with PTGG30 chains.

It additionally appears that PTGG30/Lys offers an optimal molecular weight and ease of conjugation to maintain lysozyme activity, which was also preserved during lyophilization (Figure 4B; up to 10 cycles with <20% loss of activity vs a 71% loss for the free enzyme). The better performance of PTGG-ylated over PEGylated enzymes (66–67% loss of activity) is specifically ascribed to the protective action of glycerol’s hydroxyl groups and is a highly advantageous feature: the long-term storage and distribution of these conjugate proteins would not require other cryo/lyoprotectants excipients, some of which, for example, PEG-containing polysorbate/Tweens, are known to contribute to denaturation of protein via oxidation.^54^

#### (C) Effect on Immune Recognition

The conjugation of synthetic polymers to a therapeutic protein, among other benefits, provides steric shielding of the antigenic portions of the protein, thus reducing immunogenicity and extending its half-life in vivo. For an accurate comparison of the efficacy of PTGG vs mPEG, we have assessed the capacity of antilysozyme antibodies to recognize free lysozyme, PTGG30/Lys and mPEG/Lys[1], that is, two conjugates with an analogous pattern of similarly sized polymer chains (Figure 4C). Both polymers were able to almost completely abrogate recognition by a monoclonal antibody and strongly reduced that by polyclonal antibodies; the lower shielding efficacy of the polyclonal antibodies is a consequence of the multiplicity of binding sites accessible, which statistically would also include regions less sterically hindered by the polymer chains. In summary, PTGG30 provided lysozyme with a protection from immune recognition broadly analogous to that of a similarly sized and conjugated mPEG.

#### (D) Protection from Proteolysis

**Figure 5:**
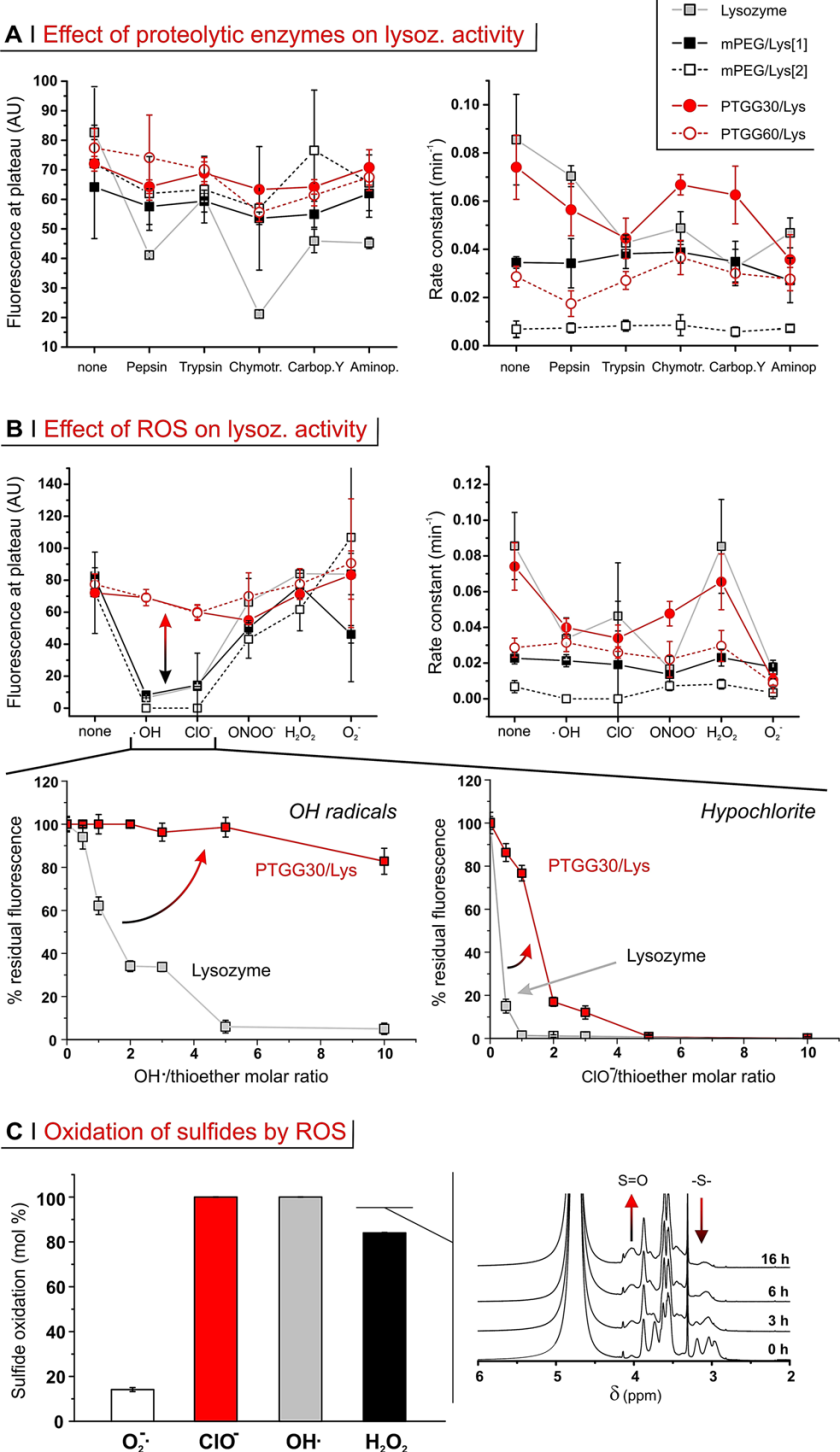
(A) Lysozyme and its derivatives (1 mg/mL) were incubated for 24 h with a panel of proteases (10 mg/mL) prior to measuring their activity and fitting their data (see also Supporting Information, Figure S5A) to extract fluorescence at plateau (left) and rate constant (right) data. (B) Top panels: activity of lysozyme and its derivatives (1 mg/mL) upon incubation with various ROS (24 h for H_2_O_2_ and H_2_O_2_/Cu(II) (OH radicals), 3 h for peroxynitrite and superoxide, and 13 min for hypochlorite; for PTGG derivatives, the oxidant/thioether molar ratios were respectively of 3:1, 5:1, 0.5:1, 3:1, and 0.5:1). Bottom panels: Normalized fluorescence at plateau upon exposure of free lysozyme and PTGG_30_/Lys to increasing amounts of OH radicals (24 h) and hypochlorite (15 min); please note that the molar ratios to thioethers (horizontal scale) refer to PTGG only. Error bars represent ± the standard deviation (*n* = 3). (C) Extent of sulfide oxidation for PTGG30/Lys upon 24 h incubation with various ROS at 10:1 oxidant/thioether molar ratio (left), as calculated from the decrease in intensity of the sulfide-associated resonance (δ ∼ 3 ppm) in ^1^H NMR spectra of PTGG in D_2_O (right: spectra obtained during incubation with H_2_O_2_.

Proteolysis is an issue for all protein-based therapeutics; this degradation is arguably more significant in the case of oral administration (gastrointestinal tract digestive enzymes), although it also is very relevant for intradermally or subcutaneously administered formulations.^24a^ Here, we have used a panel of common proteases: three endopeptidases, that is, pepsin (produced in stomach), trypsin and chymotrypsin (both pancreas), and two exopeptidases, that is, carboxypeptidase Y (acting on C-termini) and aminopeptidase (on N-termini). For this analysis, we have again employed two parameters: fluorescence at plateau (Figure 5A, left) and rate constant (Figure 5A, right).

The fluorescence at plateau (→ breadth of possible substrates, thus direct damages to/stability of the active site) of free lysozyme was strongly reduced by treatment with chymotrypsin (∼75% reduction) and to a lesser extent with pepsin and with both exopeptidases. Conversely, the conjugates remained largely unaffected, which indicates that they were all able to reduce damages directly at the active site. In terms of the effects on the rate constant (→ number and turnover rate of the active enzymes), PTGG30/Lys was more stable to chymotrypsin and carboxypeptidase Y than free lysozyme, but both were rather insensitive to pepsin and significantly affected by the remaining enzymes. The larger molecular weight PTGG_60_/Lys does display the lowest relative decreases in activity with respect to the PTGG_30_/Lys and mPEG/Lys[1], suggesting that larger molecular weights offer greater steric protection from protease-mediate degradation; however, its absolute rate constant remained low because of the conjugation of PTGG60 itself. We further refrain from making strong assessment on the more highly conjugate mPEG/Lys[2] for similar reasons.

#### (E) Protection from ROS

Thioethers (sulfides) may react differently with different oxidants. For example, while hydrogen peroxide commonly stops at the level of sulfoxides, hypochlorite proceeds also further to sulfones, which then can spontaneously decompose causing chain fragmentation/depolymerization;^22b^ at the same time, polysulfides appear to respond poorly to superoxide.^55^ Here, we have examined the effects of a panel of physiologically relevant oxidants on lysozyme and lysozyme-polymer conjugates (Figure 5B).

The fluorescence at plateau showed free lysozyme activity to be heavily hampered by hypochlorite (ClO^–^), hydroxy radicals (^·^OH), and—to a lesser extent—peroxynitrite (ONOO^. -^) and superoxide (O_2_^·–^) (Figure 5B, top left). The same four ROS also had detrimental effects on the rate constant (Figure 5B, top right). On the contrary, both kinetic parameters indicated that lysozyme was insensitive to the presence of H_2_O_2_ (even up to 54 mM; see Supporting Information, Figure S6C).

Both PEG derivatives closely tracked unconjugated enzyme in terms of fluorescence at plateau; this indicates that PEG chains did not offer any significant protection of the active site from oxidants. Please note that PEGylation of Lys/PEG[2] decreased the rate constant to such an extent that this parameter could not be considered a sufficiently sensitive indicator, as previously seen for proteolysis.

Differently from PEG, PTGG chains clearly protected lysozyme activity from the action of ROS (see Supporting Information, Figure S5B). This effect was particularly noticeable in the protection of the active site from hydroxy radicals and hypochlorite (double-headed arrow in Figure 5B, top left). Indeed, ·OH and ClO^–^ reduced the fluorescence at plateau in mPEG/Lys[1] (similarly for free lysozyme) respectively by ∼90 and ∼80%, whereas PTGG30/Lys only experienced a modest 15% reduction. Interestingly, the mechanism of this protective action appears to be different for the two ROS (Figure 5B, bottom panels); for hypochlorite, the concentration dependency points toward a stoichiometric scavenging of the ROS by sulfur atoms, while a much wider range of inhibition of hydroxy radicals may rather suggest a form of catalytic mechanism. It is worth pointing out that the polysulfide chains did not have any noticeable effect in the presence of superoxide (see Supporting Information, Figure S6D). With H_2_O_2_ PTGG sulfides are quantitatively oxidized—similarly to what happens with hydroxy radicals and hypochlorite (Figure 5C)—but this scavenging does not provide additional protection, because lysozyme is inherently stable to hydrogen peroxide. For superoxide, on the contrary, the lack of protection (see also Supporting Information, Figure S6D) is simply because polysulfides demonstrated low capacity to scavenge it^55^ and to thus be oxidized by it (white bar in Figure 5C). In summary, the conjugation of (short) PTGG chains was shown to well preserve enzymatic activity also under harsh proteolytic and oxidizing conditions, typically better than an equivalent amount (in number and size) of PEG.

### Pharmacokinetics of PTGG Conjugates

#### (A) Characterization of OVA Conjugates

**Figure 6:**
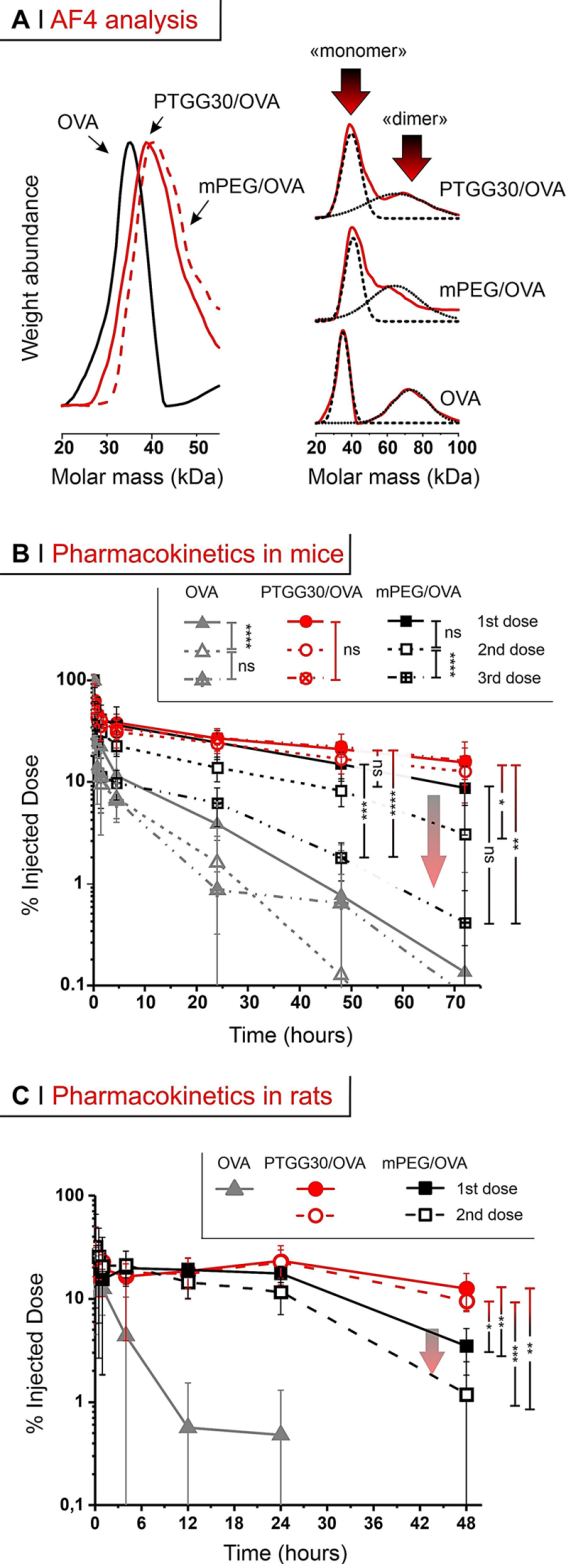
(A) Left: molecular weight distributions obtained via AF4 (refractive index and static light scattering detectors). Right: the initial OVA and its conjugates have a bimodal distribution, not seen under the reducing conditions used in SDS-PAGE (Figure S4B). (B) % of ID in blood after tail vein injection at 500 μg/kg of OVA in mice; arrow highlights the ABC effect. (C) As in B, after tail vein injection in rats. A two-way and a one-way (with Tukey’s means comparison) ANOVA were respectively used to assess significance within treatment groups at different doses (legend) and between groups at 48 and 72 h; *P ≤ 0.05, **P ≤ 0.01, ***P ≤ 0.001, ****P ≤ 0.0001.

For an in vivo assessment, we have chosen a larger and more immunogenic protein (ovalbumin, OVA) over lysozyme. OVA is sufficiently immunogenic to stimulate an ABC effect in its PEGylated conjugates,^39^ which therefore allows for a more complete PEG/PTGG comparison. Furthermore, OVA is also larger than lysozyme (42–47 kDa vs 16 kDa), which significantly reduces the chance of its conjugates to undergo renal filtration; this allows therefore to ascribe its elimination from the blood stream to a more active biological clearance (e.g., immune capture/degradation). OVA was thus conjugated with 5 kDa PEG or 5 kDa PTGG (see the molecular weight distribution shifting to larger values in Figure 6A, left); of note, PEGylation with 5 kDa PEG is FDA-approved and clinically used in, for example, Asparlas, Oncaspar, Adagen, and Somavert.

Field-flow fractionation analysis (in asymmetric flow mode, AF4) showed OVA to have a bimodal distribution, with a ‘monomer’ and a ‘dimer’ peak, which remain present after conjugation (Figure 6A, right); the average mass calculated on the whole distribution is however in line with literature data. The degree of conjugation was assessed via A4F, SDS-PAGE, and the analysis of amine consumption (see Supporting Information, Figure S7B and Table S1) and indicated an average of 2–3 chains per OVA.

#### (B) Conjugate Pharmacokinetics in Mice

In mice, the circulating dose of free OVA (gray triangles in Figure 6B) after tail vein injection was reduced to less than 5% of the injected dose (ID) in 24 h upon first injection.

Clearance was more efficient upon repeated administration (second dose at day 7, third dose at day 14, see a schematic timeline in Supporting Information, Scheme S1, left): for example, at 24 h after the 3rd administration, OVA’s remaining dose was about 1% of the ID.

Both PEG- and PTGG-OVA conjugates displayed a rapid first/α-phase blood clearance of up to ∼40% in the first 15 min; this phase is common upon parenteral administration of PEGylated nanostructures in vivo, as seen in mice,^56^ in rats^57^ and even in humans.^58^ Of note, this rapid first (α-) phase of elimination was in any case much less intense and slower than that of OVA (see Supporting Information, Table S2), a phenomenon already seen in mice for PEGylated OVA.^39^ PEGylation (black squares in Figure 6B) also considerably prolonged the protein’s long-term circulation (β-phase of elimination), but with a clear ABC effect: 8–9% of the ID was still present at 72 h post-1st dose, but this decreased to about 0.5% at 72 h post-3rd dose. The PTGG OVA conjugate (red symbols in Figure 6B) showed an almost 50% longer circulation than the PEG conjugate after the 1st dose (*t*_1/2β_ respectively = 31.7 and 48.9 h; Table 3); interestingly, this difference is similar to what is recorded in head-to-head comparisons of polyglycerols and PEG, where the former exhibited *t*_β1/2_ about 1.5–2× longer than PEG.^30^ Most importantly, PTGG showed no ABC effect: for example, PTGG/OVA at 72 h had still 15% of the ID, independent of the number of doses, with a 5-time longer *t*_1/2β_ than PEG/OVA on its third dose.

**Table 3:**
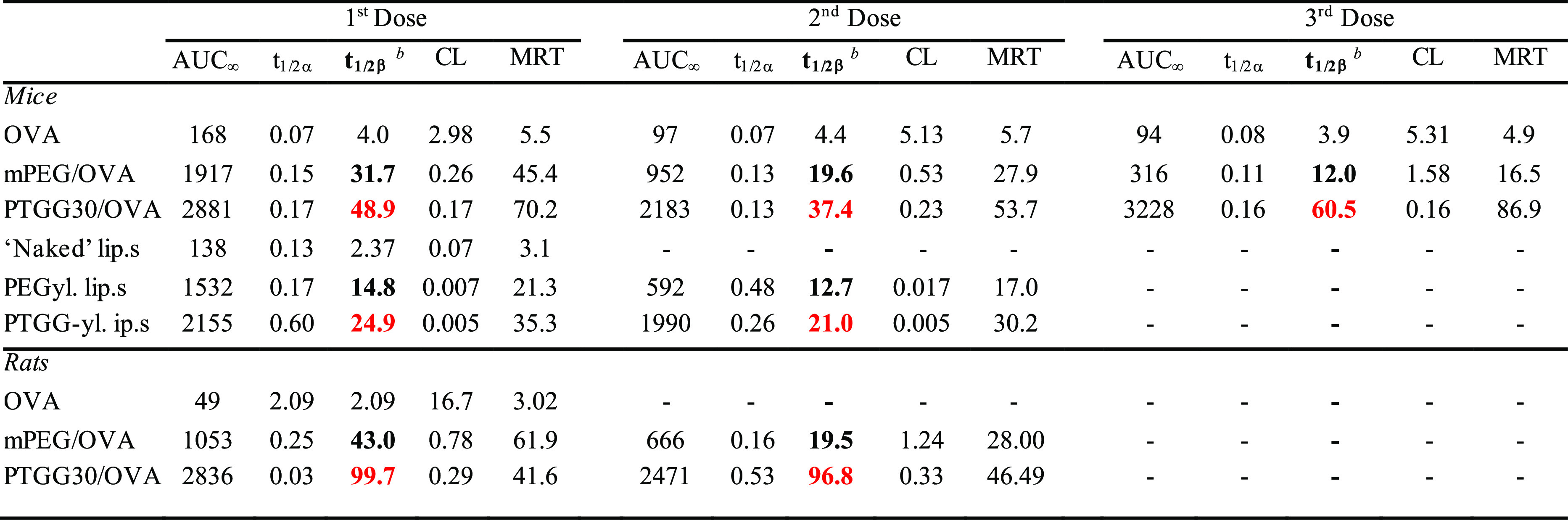
Summary of the Noncompartmental Pharmacokinetic Data^a^

a Averages over *n* = 4 for conjugates and liposomes, *n* = 3 for OVA. Units: AUC – μg/mL h; *t*_1/2α_, *t*_1/2β_ – h; CL (clearance rate) – mL/h; MRT (mean residence time) – h.

b In bold the comparison between long-term half-life time of PEGylated (black) and PTGG-ylated (red) constructs.

**Figure 7:**
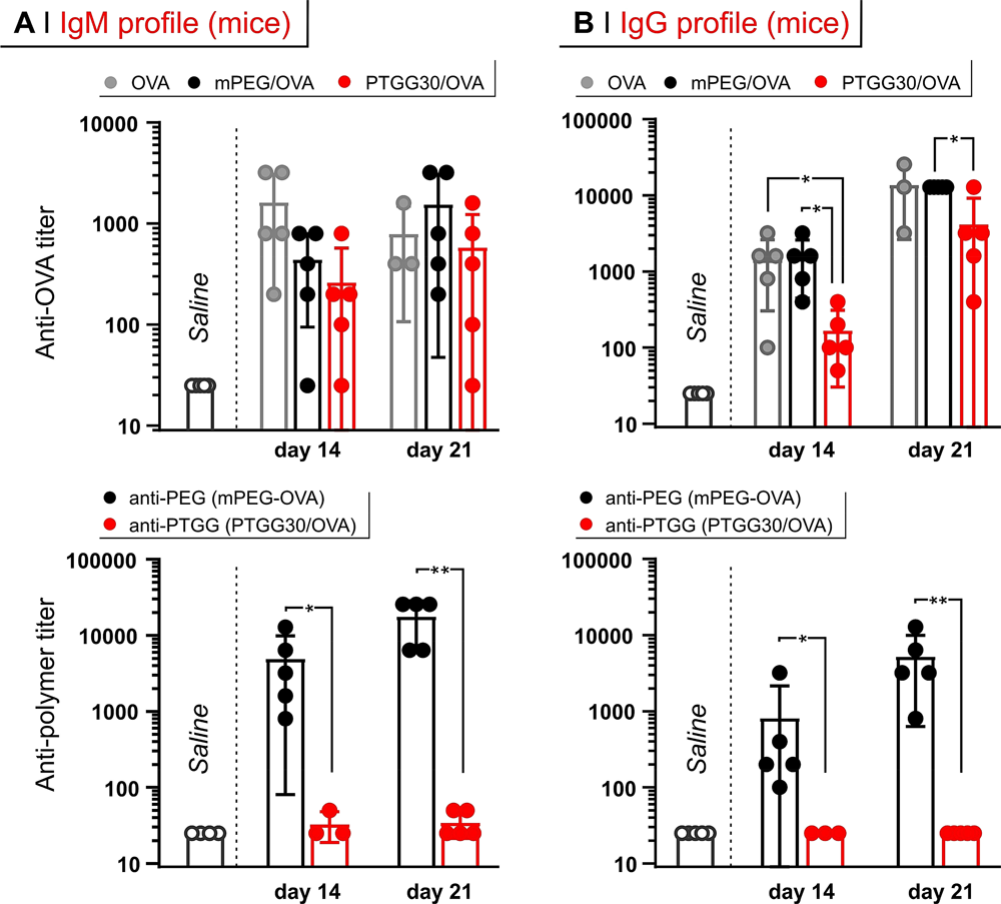
Immunogenicity of OVA, PEG/OVA, or PTGG/OVA was quantified via their capacity to induce the production of IgM (A) and IgG (B) against OVA (top) or the two synthetic polymers (bottom), as measured via ELISA on mice sera collected on either day 14 (animals dosed at days 1 and 7) or day 21 (dosed at days 1, 7, and 14). Direct ELISA was not performed on PEG/OVA or PTGG/OVA conjugates due to the low level of plate adhesion for each of these; for a more extensive explanation/analysis, see Supporting Information, Figure S9). To test for significance, a one-way ANOVA with a Tukey’s means comparison test at days 14 and 21 was performed for anti-OVA titers, and a Mann–Whitney test was used to assess antipolymer titers; *P ≤ 0.05; **0.001 ≤ *P* ≤ 0.01.

Interestingly, the different pharmacokinetic behavior of the conjugates mirrored their different immunogenicity (Figure 7). In terms of the protein cargo, anti-OVA IgG and IgM titers were indistinguishable with or without PEGylation, although the latter are likely to have a lower affinity or are totally blocked (sterically) from their OVA epitope by PEG chains (see lysozyme, Figure 4C), hence longer circulation times than free OVA.

PTGG on the one hand significantly reduced or delayed the anti-OVA IgG response (i.e., IgM class switching), with possibly some effects also on IgMs production also (at day 14, not significant). They also did not elicit any measurable production of IgM or IgG anti-PTGG antibodies which in comparison to PEG/OVA stimulated a large presence of both anti-PEG IgMs (Figure 7A, bottom) and IgGs (Figure 7B, bottom), which increased with the number of doses. Therefore, the more prolonged circulation of PTGG/OVA may derive from a combination of a more efficient immune protection of the cargo with the lack of a direct adaptive immunogenic response of the polymer. Among the possible interpretations, this effect may stem from the ubiquitous etabolic presence of glycerol and its derivatives, which would make it difficult to mount a humoral response against PTGG as it is possibly too similar to ‘self’-antigens.

Another possibility invokes the peculiarity of the diol in glyceryl derivatives, where the OH groups can form intramolecular H bonds more easily than e.g., in ethylene glycol; this would on the one hand provide a low nucleophilicity as in PEG, and on the other hand increase the hydrophilicity and water solvation around the macromolecule. Finally, the potential oxidizability of PTGG (e.g., sulfide, sulfoxide, and sulfone) may also make it particularly challenging to mount a coordinated humoral response against the backbone due to the variable ratio between reduced and oxidized-PTGG epitopes. However, because of the lack of the ABC effect seen in other polyglycerols lacking a sulfide backbone, we believe that the former explanations are more likely.

#### (C) Conjugate Pharmacokinetics in Rats

We have confirmed the above pharmacokinetic results using a different animal model (rats), in order to show that the PTGG advantages are not species-specific (Figure 6C). Upon tail vein injection, unconjugated OVA was rapidly cleared (6–8 h) as in mice; after a single (1st) dosing of PEG- or PTGG-OVA conjugates, both experienced a rapid α-phase (down to ∼20–32% of the ID, slightly less than in mice), followed by a slower β-component of clearance (down to 17.5 and 23.1% ID after 24 h respectively). PTGG, however, maintained a higher concentration (e.g. ∼13% ID after 48 h) than PEG (3.5% ID), with a ∼ 2× longer *t*_1/2β_ (99.7 vs 43.0 h). Upon a second dose in rats, and similarly to what was seen in mice: PEG-OVA clearance was significantly hastened upon a second injection both in the α- (*P* = 0.002 at 0.15 and*P* = 0.0234 at 0.5 h) and in the β-phase, where t_β1/2_ was reduced from 43 to 19.5 h (Table 3). This tallies with existing literature, which shows the ABC effect of PEG-protein conjugates to occur at a similar degree in rats^19^ and mice.^39^ On the contrary, no significant difference between PTGG-OVA first and second dose clearances was recorded in any of the phases. For example, *t*_1/2β_ was 99.7 h in the first administration and 96.8 h in the second, and no significant difference can be noticed also in the area under the curve (AUC). Also here, this is in line with literature reports showing that if synthetic polymers such as polyglycerols lack an ABC, they do so both in mice^31a^ and rats^31b^

#### (D) Pharmacokinetics of Liposomes in Mice

To have a more complete overview of the capacity of PTGG to act as a ‘stealth’ modifier, we have prepared a PTGG-containing lipid (a derivative of dipalmitoyl phosphoethanolamine; for a structure, refer to Supporting Information, Figure S2) and used it to produce PTGG-ylated liposomes. The latter contained also 61 and 33 mol % resp. of l-α-phosphatidylcholine and cholesterol and were compared with analogous ‘naked’ and PEGylated structures.

**Figure 8:**
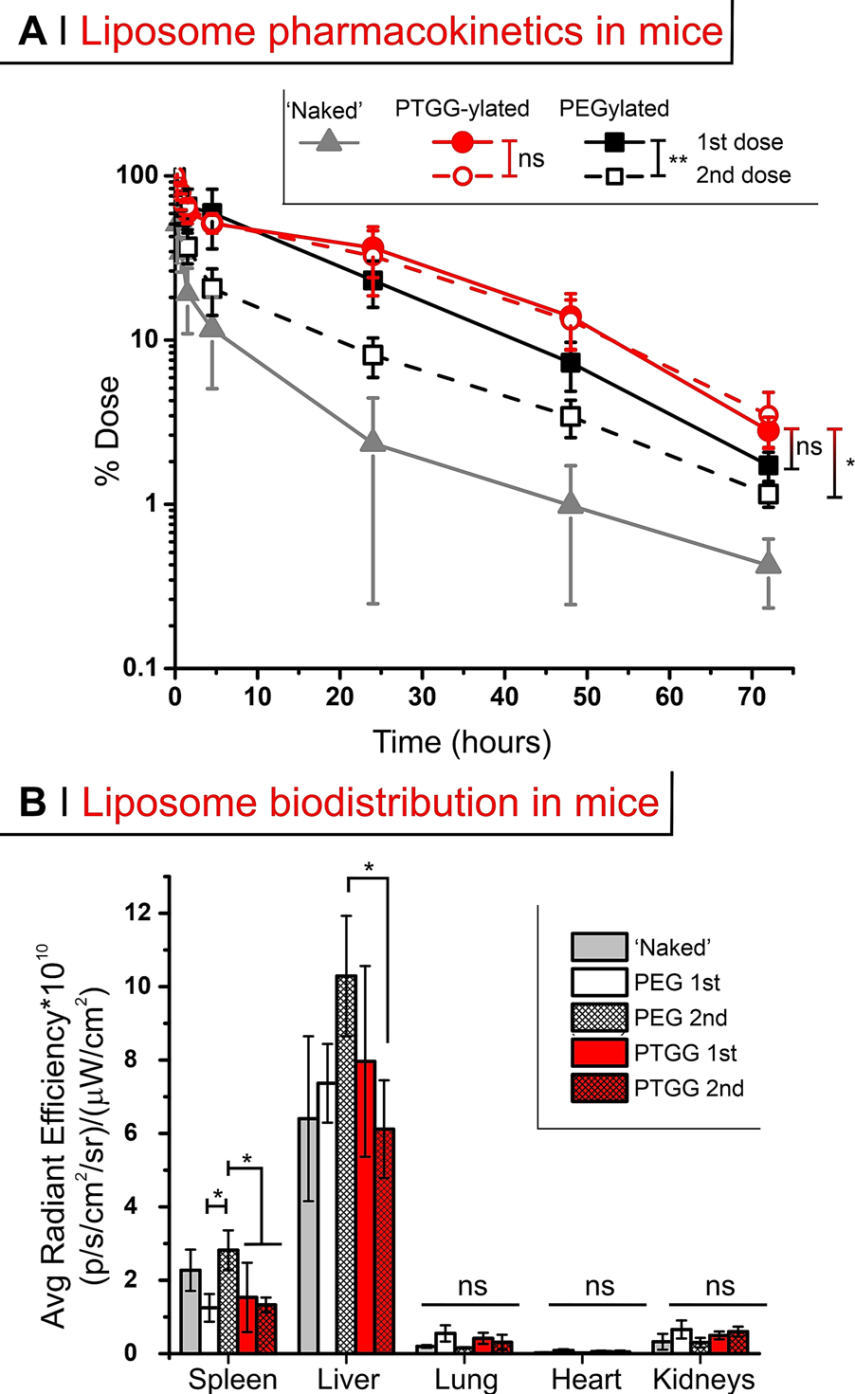
(A) Pharmacokinetics of ‘naked’-, PEGylated-, or PTGG-ylated liposomes after first (day 1) or second (day 7) injection. (B) Biodistribution of the liposomes as assessed by measuring the fluorescence of DiD in the different organs (see Supporting Information, Figure S9). A two-way ANOVA was used to assess statistical significance within treatment groups at different doses (legend) whereas a one-way ANOVA with a Tukey’s means comparison test was used to assess statical significance between groups at 72 h; *P ≤ 0.05; **0.001 ≤ *P* ≤ 0.01.

At a single dose, PTGG-ylated liposomes displayed a 1.7× longer *t*_1/2β_, and a 1.4× larger AUC than their PEGylated counterparts (Figure 8A). More importantly, in a double-dose regime, even days after the second injection PTGG-ylated liposomes retained nearly identical pharmacokinetic parameters, whereas PEGylated liposomes showed a much reduced residual ID at all time points, and underwent a 2.6× reduction in AUC, both being evidences of ABC. All the different liposomes showed accumulation in liver (main) and spleen (secondary), but it was only the second dosed PEGylated liposomes that showed a significantly higher uptake in those organs (Figures 8B and S9), which is again a strong indication of the ABC effect being operational for PEG but not for PTGG.

In summary, the long circulation times and the apparent absence of an ABC effect favorably combine with the previously shown advantageous characteristics (anti-inflammatory, cryo/lyoprotectiveness, low toxicity, reduced complement activation, and lack of antibody recognition), indicating PTGG as a promising alternative to traditional stealth polymers that can trigger ABC effects; this is particularly important for therapeutics that require multiple doses.

## Conclusions

This report presents a macromolecular structure (PTGG) suitable for bioconjugation, which on one hand can enhance physical stability and reduce immunogenicity of model proteins, but on the other hand protect them from oxidative and by extension, inflammatory damage. PTGG therefore combines in its structure the behavior of a ‘stealth’ (such as PEG) and of a ‘smart’ polymer.

In reference to the ‘stealth’ polymer behavior, we have compared PTGG performance with that of molecular-weight-matched PEG, showing that PTGG was similar (or better) in toxicity, macrophage uptake, and complement activation. Furthermore, PTGG lysozyme conjugates better retained enzymatic activity and showed enhanced stability to freeze-drying, oxidation and proteolytic degradation as well as reduced immunogenicity (lower antibody binding and lower alternative compliment activation) and potent anti-inflammatory properties. These are highly advantageous properties for biological drugs which typically require large amounts of cryo/lyoprotective and antioxidant excipients to keep stable during storage.

Finally, we have evaluated PTGG’s stealth properties in three in vivo models: PTGG/OVA in mice and rats, as well as PTGG-liposomes in mice. The PTGG/OVA β-phase half-life was ∼12–48× that of the parent protein and 1.5× (mice) to 2× (rats) that of mPEG/OVA. Upon a second or third dose 7 or 14 days later, respectively, PTGG30/OVA was able to maintain its treatment naive *t*_1/2β_, unlike PEG-OVA, which displayed a 40–50% (2nd dose) and 80% (3rd dose) reduction. Analysis of mice sera confirmed a complete lack of anti-PTGG IgM and IgG antibodies, even after 3 doses of PTGG/OVA whereas sera from mice treated with PEG/OVA displayed extremely high titers of both IgM and IgG anti-PEG antibodies. A similar trend indicative of an ABC phenomena in PEGylated-but not PTGGylated-liposomes was observed, thereby suggesting that the immunological advantages of PTGG extend also to nanocarriers. In summary, these data strongly indicate an apparent absence of an ABC-like effect for PTGG.

Therefore, PTGG has demonstrated significant advantages over the current benchmark PEG, by combining an already better ‘stealth’ behavior with antioxidant, anti-inflammatory, and cryo/lyoprotective properties, which may be particularly beneficial for (oxidation-)sensitive proteins, for example, galectin-1.^25^ The latter may also lend to synergic outcomes, where the therapeutic action of a protein may be potentiated by the anti-inflammatory effects arising from the PTGG scavenging of ROS. Finally, although predominantly evaluated in the context of protein conjugates, our liposomal data would indicate that PTGG can be seen as a general platform for enhancing virtually any translationally important therapeutic.

## Supporting Information Available

All experimental section and additional data about cell uptake, enzyme stability, and molecular characterization (PDF)
