# Supplementary material for: A Reactive Oxygen Species-Scavenging ‘Stealth’ Polymer, Poly(thioglycidyl glycerol), Outperforms Poly(ethylene glycol) in Protein Conjugates and Nanocarriers and Enhances Protein Stability to Environmental and Biological Stressors
Source: J Am Chem Soc. 2022 Nov 11;144(46):21304–17. doi: 10.1021/jacs.2c09232 (PMC9706570; doi:10.1021/jacs.2c09232)
Supplement: Supplementary file 1 — ja2c09232_si_001.pdf [file ja2c09232_si_001.pdf]

A ROS-scavenging 'stealth' polymer, poly(thioglycidyl glycerol)(PTGG), outperforms PEG in protein conjugates and nanocarriers, and enhances protein stability to environmental and biological stressors.

Richard d'Arcy[1, 2, 3, #, *], Farah El Mohtadi[2, †, #], Nora Francini[1,3], Carlisle R. DeJulius[3], Hyunmoon Back[4,5], Arianna Gennari[1], Mike Geven[1], Maria Lopez-Cavestany[3], Zulfiye Yesim Turhan[1,2], Fang, Yu[3], Jong Bong Lee[4], Michael R. King[3], Leonid Kagan[4,5], Craig L. Duvall[3], Nicola Tirelli[1, 2*]

1 Laboratory for Polymers and Biomaterials, Fondazione Istituto Italiano di Tecnologia, 16163 Genova, Italy

2 Division of Pharmacy and Optometry, School of Health Sciences, University of Manchester, Oxford Road, Manchester, M13 9PT, United Kingdom

3 Department of Biomedical Engineering, Vanderbilt University, Nashville, TN 37235, USA

4 Department of Pharmaceutics, Ernest Mario School of Pharmacy, Rutgers, The State University of New Jersey, 160 Frelinghuysen Road, Piscataway, NJ 08854, USA.

5 Center of Excellence for Pharmaceutical Translational Research and Education, Ernest Mario School of Pharmacy, Rutgers, The State University of New Jersey, 160 Frelinghuysen Road, Piscataway, NJ 08854, USA.

Supporting information

S1 Materials and physico-chemical methods

S1.1 Materials

Chemicals. All chemicals were used as received from suppliers unless otherwise stated. Epichlorohydrin, 1,2-isopropylidene glycerol (solketal), thiourea, ammonium chloride (NH_4Cl), S-phenyl thioacetate, 2-bromo-1,1-dimethoxyethane, sodium methoxide (NaOMe, 0.5 M in methanol), tris(hydroxymethyl phosphine), 1,8-diazabicyclo[5.4.0]undec-7-ene (DBU), sodium bicarbonate ($NaHCO_3$), potassium hydroxide (KOH), anhydrous sodium sulfate (Na_2SO_4), sodium thiosulfate, potassium iodide, anhydrous magnesium sulfate ($MgSO_4$), triphenyl phosphine, diisopropyl azodicarboxylate (DIAD), thioacetic acid, PEG monomethyl ether ($\overline{M_n}$ ≈ $5*10^3$ g/mol), FITC–labelled cationic dextran (FITC–DEAE–Dextran, Mw = 3-6 kDa), Dowex 50WX8, ovalbumin from chicken egg white (lot No SLCH2414, OVA), hen egg white lysozyme and copper(II) chloride were purchased from Sigma-Aldrich (Gillingham, UK). β-maleimidopropionic acid N-hydroxysuccinimide ester (BMPS) was purchased from Fluorochem (Hadfield, UK). 3-((3-cholamidopropyl) dimethylammonio)-1-propanesulfonate (CHAPS), dichloromethane, diethyl ether, 1,1'-dioctadecyl-3,3,3',3'-tetramethylindodicarbocyanine 4-chlorobenzenesulfonate (DiD), dioxane, tetrahydrofuran (THF), N,N-dimethyl formamide (DMF), methanol, hydrochloric acid 1 M (HCl), were purchased from Thermo Fisher Scientific (Leicestershire, UK). Cholesterol, 1,2-dipalmitoyl-sn-glycero-3-phosphoethanolamine-N-[methoxy(polyethylene glycol)-5000] ammonium salt (16:0 PEG5000 PE), 1,2-dipalmitoyl-sn-glycero-3-phosphoethanolamine-N-[4-(*p*-maleimidomethyl)cyclohexane-carboxamide] sodium salt (16:0 PE MCC) and hydrogenated egg L-α-phosphatidylcholine (HEPC) were purchased from Avanti Polar Lipids (Birmingham, USA).

Cells, assays and buffers. Neonatal human dermal fibroblasts (HDFn) were purchased from Thermo Fisher (Leicestershire, UK). Fetal bovine serum (FBS), human serum, glutamine, antibiotics (penicillin/streptomycin), ascorbic acid, Tween 80, Triton X-100, β-glycerophosphate disodium salt hydrate (BGP) and Dulbecco's modified eagle medium (DMEM), lipopolysaccharides (LPS) from Escherichia coli 0111:B4 were all purchased from Sigma-Aldrich (Gillingham, UK). The MTS proliferation assay (CellTiter 96® Aqueous One Solution Cell Proliferation Assay) was purchased from Promega (Chilworth, UK) (Leicestershire, UK). Phosphate buffered saline (PBS) Dulbecco A tablets were purchased from Oxoid (Hampshire, UK). SIN-1 chloride, chymotrypsin, and carboxypeptidase Y and

zymosan from Sigma-Aldrich (Gillingham, UK), pepsin and potassium superoxide were purchased from Acros Organics (Geel, Belgium) and H_2O_2 30% from VWR (Radnor, USA). Trypsin and NaOCl (10-14%) were purchased from Alfa Aesar (Heysham, UK) .Note: H_2O_2 and NaOCl were titrated to determine true concentration prior to use. C3a and C5a Platinum ELISA kits were purchased from eBioscience (San Diego, USA), Mouse TNF-α ELISA kit was purchased from Cohesion Bioscience (London, UK) and the Hypochlorite Assay (colorimetric, abs. 555 nm) and Hydrogen Peroxide Assay (abs. 535 nm) were both purchased from Mito Sciences (Cambridge, UK). For 'homemade' ELISAs, a 3,3',5,5'-tetramethylbenzidine (TMB) ELISA Substrate (High Sensitivity) solution (ab171523) purchased from Abcam (Cambridge, UK) was used.

S1.2 Physico-chemical characterization

^{1}H NMR spectroscopy. Spectra were recorded on 1.5% wt. solutions in deuterated dimethyl sulfoxide-d6 (DMSO), chloroform ($CDCl_3$), or water (D_2O) using a Bruker Avance 300 or 400 MHz Bruker spectrometer.

FT-IR spectroscopy. Spectra were recorded in ATR mode (Golden Gate) on a Tensor 27 Bruker spectrometer (Bruker UK Limited, UK) equipped with a 3000 Series TM High Stability Temperature Controller with RS232 Control (Specac, UK).

Gel permeation chromatography (GPC). Molecular weight distributions were obtained using a triple detection set up of the Polymer Laboratories PL-GPC50 integrated GPC (Polymer Laboratories, UK) comprising a Plgel 5 μm Guard, two Polypore 5 μm columns operating online at 35 °C with a THF eluent at a flow rate of 1.0 mL/min. Calibration was performed using polystyrene standards of known molecular weight, intrinsic viscosity and dn/dc.

Fluorescence and UV-Vis spectroscopy. Readings were obtained through a BioTek Synergy 2 multimode microplate reader (BioTek, U.K.) at a temperature of 37 °C.

Asymmetric Flow Field-Flow Fractionation (AF4). An AF4 system AF2000 TM (Postnova Analytics, Landsberg, Germany) was coupled online to a PN3609 multi-angle light scattering (MALS) (Postnova Analytics, Landsberg, Germany) and a PN3150 refractive index (Postnova Analytics, Landsberg, Germany) detector in the given order. The AF4 channel was equipped with a 350 μm spacer and a 10 kDa MWCO membrane of regenerate cellulose as accumulation wall. 0.1 μm-filtered PBS was used as eluent. The data collected by the MALS and refractive index detectors were analyzed with an AF2000 software (Postnova Analytics) and fitted with a Debye model to obtain the molecular weight

considering dn/dc = 0.185 mL/g for all samples. In a typical experiment, the detector flow rate was set at 0.5 mL/min and 50 µL of a 2.5 mg/mL sample solution in PBS were injected over 4 minutes setting 0.2 mL/min as injection flow rate. For the elution step the cross flow was maintained constant at 4 mL/min for 20 min and then linearly decreased to 0 mL/min over 5 minutes, subsequently keeping at 0 mL/min for additional 5 minutes.

Dynamic Light Scattering (DLS). Liposome samples were prepared at 0.5 µmol/mL of phospholipid, spiked with a small amount of sodium hypochlorite to bleach DiD, and measured using a Malvern Zetasizer Ultra.

S1.3 Preparative procedures

S1.3.1 Monomer synthesis

Synthesis of glycidyl solketal (GS).

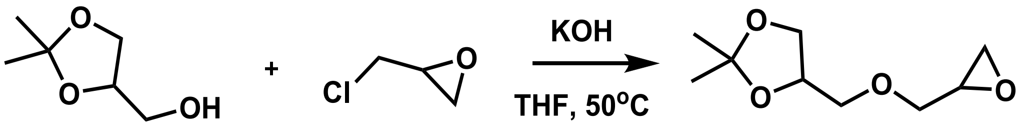

In a three-necked round bottom flask, 40 g (303 mmol) of 2-isopropylideneglycerol and 71 mL of epichlorohydrin (908 mmol, 3 equiv.s) were dissolved into 150 mL of THF under argon. The flask was placed in an ice bath, adding 20.4 g of potassium hydroxide pellets (363 mmol, 1.2 equiv.s) previously crushed in a mortar. The reaction was kept at 0 °C for 3 hours, then leaving the reaction at room temperature overnight and finally heating it to 50 °C for 2 hours. After cooling to ambient temperature, insoluble materials were filtered and the solution concentrated via rotary evaporation. The residue was diluted with dichloromethane (200 mL) and washed with 3x50 mL of 10 w/v% of sodium bicarbonate ($NaHCO_3$) followed by 2x50 mL of brine. The organic phase was separated, dried over Na_2SO_4, filtered and concentrated *in vacuo*. Vacuum distillation was used to obtain 28 g of the product as colorless oil (70% yield).

^{1}H NMR ($CDCl_3$): δ = 1.36, 1.43 (ss, 6H, -O-C(C**H**$_3$)$_2$-O-, ketal methyls), 2.59-2.62 (m, 1H, 1 diastereotopic **H** of epoxide CH$_2$ (-O-C**H$_2$**-)CH-CH$_2$-O-, 2.78-2.81 (t, 1H, 1 diastereotopic **H** of epoxide CH$_2$ (-O-C**H$_2$**-)CH-CH$_2$-O-), 3.13-3.18 (m, 1H, epoxide CH (-O-CH$_2$-)C**H**-CH$_2$-O- 3.38-3.86 (m, 5H, 1 diastereotopic *H* of C*H$_2$* (italicized) -C**H**$_2$-O-C**H**$_2$-CH(-C***H$_2$***-O-C(CH$_3$)$_2$)-O-)), 4.04-4.08 (t, 1H, 1 diastereotopic **H** of CH$_2$ - -O-CH$_2$-CH(-C**H**$_2$-O-C(CH$_3$)$_2$-O-)), 4.24-4.34 ppm (m, 1H, -O-CH$_2$-C**H(**-CH$_2$-O-C(CH$_3$)$_2$-O-)).

ATR FT-IR (thin film): 2988 (ν_{as} CH_3), 2932 (ν_{as} CH_2), 2875 (ν_s CH_2), 1450 (δ CH_2), 1369 (δ CH_3), 1147 (ν_{as} C-O-C), 1086 (ν_s C-O-C), 1050 cm^{-1} (ν_{as} C-O-C).

Synthesis of thioglycidyl solketal (TGS).

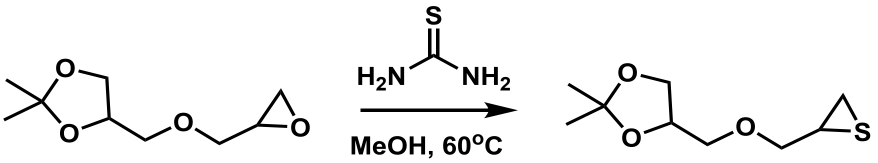

7.5 g (40 mmol) of GS, 6.1 g (80 mmol, 2 equiv.s) of thiourea and an excess (23 g) of NH_4Cl were dissolved in 100 ml of methanol and introduced into a three-necked round bottom flask. The reaction mixture was then heated at 60 °C, following the reaction via TLC using dichloromethane as the eluent (disappearance of band at RF=0.35, appearance of new band at RF=0.75). After reaction completion, methanol was removed under reduced pressure and the material dissolved in dichloromethane (150 mL) in the presence of water (100 mL). The organic phase was separated and extracted with 50 mL of water twice more. The organic phase was finally dried over Na_2SO_4, filtered and concentrated under vacuum. The resulting oil was columned-filtered through silica using a 3:1 hexane:dichloromethane mixture to yield 6.8 g of colorless oil (90 % yield).

^{1}H NMR (DMSO); δ = 1.26 (s, 3H, C**H**$_3$-C(CH$_3$)-O-CH), 1.31 (s, 3H, C**H**$_3$-C(CH$_3$)-O-CH), 2.29-2.31 (d, 1H, 1 diastereotopic **H** of episulfide CH$_2$ (-S-C**H**$_2$-)CH-CH$_2$-O-, 2.55-2.57 (d, 1H, 1 diastereotopic **H** of episulfide CH$_2$ (-S-C**H**$_2$-)CH-CH$_2$-O-, 3.09-3.17 (m, 1H, (-S-CH$_2$-)C**H**-CH$_2$-O-, 3.46-3.63 (m, 5H, 1 diastereotopic ***H*** of CH$_2$ (italicized) –C**H**$_2$-O-C**H**$_2$-CH(-C***H***$_2$-O-C(CH$_3$)$_2$-O-)), 3.96-4.01 (q, 1H, 1 diastereotopic **H** of CH$_2$ - -O-CH$_2$-CH(-C**H**$_2$-O-C(CH$_3$)$_2$-O-)), 4.14-4.22 ppm (p, 1H, -O-CH$_2$-C**H(**-CH$_2$-O-C(CH$_3$)$_2$-O-).

^{1}H NMR (CDCl$_3$); δ = 1.38 (s, 3H, C**H**$_3$-C(CH$_3$)-O-CH), 1.44 (s, 3H, C**H**$_3$-C(CH$_3$)-O-CH), 2.22-2.23 (d, 1H, 1 diastereotopic **H** of episulfide CH$_2$ (-S-C**H**$_2$-)CH-CH$_2$-O-, 2.53-2.55 (d, 1H, 1 diastereotopic **H** of episulfide CH$_2$ (-S-C**H**$_2$-)CH-CH$_2$-O-, 3.05-3.13 (m, 1H, (-S-CH$_2$-)C**H**-CH$_2$-O-, 3.49-3.78 (m, 5H, 1 diastereotopic ***H*** of CH$_2$ (italicized) –C**H**$_2$-O-C**H**$_2$-CH(-C***H***$_2$-O-C(CH$_3$)$_2$-O-)), 4.06-4.11 (q, 1H, 1 diastereotopic **H** of CH$_2$ - -O-CH$_2$-CH(-C**H**$_2$-O-C(CH$_3$)$_2$-O-)), 4.26-4.35 ppm (p, 1H, -O-CH$_2$-C**H(**-CH$_2$-O-C(CH$_3$)$_2$-O-).

ATR FT-IR (thin film): 2983 (ν_{as} CH_3), 2929 (ν_{as} CH_2), 2869 (ν_s CH_2), 1455 (δ CH_2), 1372 (δ CH_3), 1149 (ν_{as} C-O-C), 1086 (ν_s C-O-C), 1045 (ν_{as} C-O-C), 613 cm^{-1} (ν C-S in TGS).

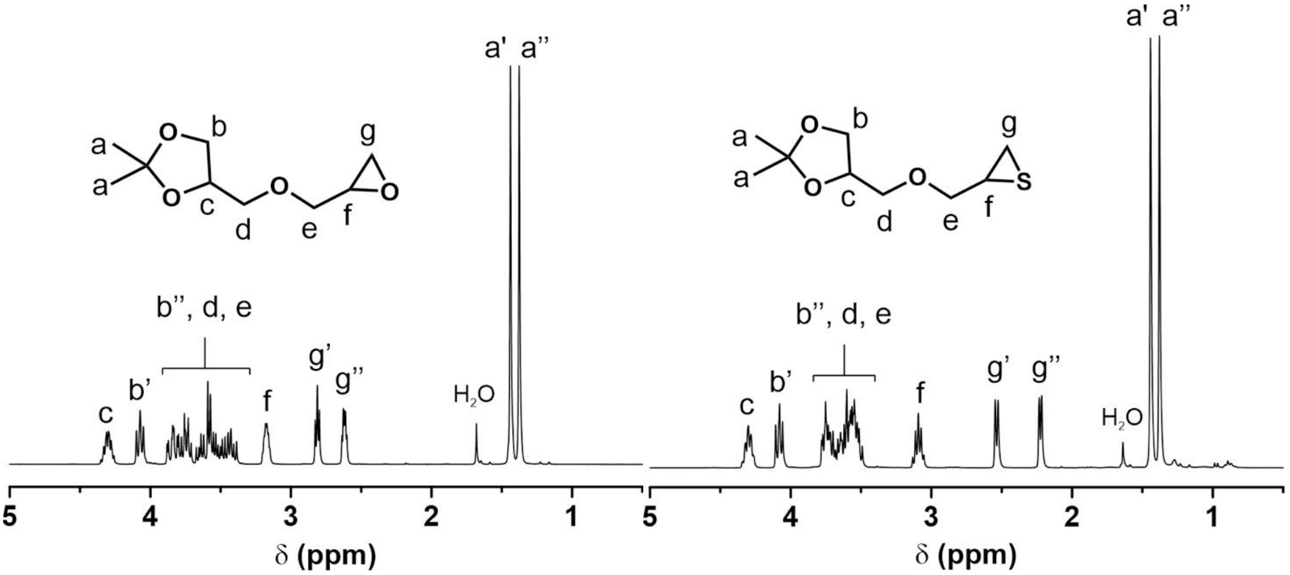

Figure S1. ^{1}H NMR spectra of glycidyl solketal (left) and of thioglycidyl solketal (right) in $CDCl_3$; letters correspond to the protons as assigned in the chemical structure.

S1.3.2 Polymer synthesis and bioconjugation reactions

Synthesis of poly (thioglycidyl solketal) (PTGS). All experiments were conducted using a 12-position Carousel parallel reactor (Radleys, Saffron Walden UK) which was purged with argon 10 minutes prior to use. 5 mL of degassed DMF (argon bubbling for 1 hr) were transferred in each position of the reactor with 24.4 mg (0.16 mmol) of S-phenyl thioacetate followed first by 101.2 mg of tris (hydroxymethyl) phosphine (THMP) (0.81 mmol, 5 equiv.s) and then by 0.39 mL of a 0.5 M solution of sodium methoxide in methanol (0.19 mmol, 1.05 equiv.s). After 10 min, 14.7 mg of acetic acid (0.24 mmol, 1.5 equiv.s) were added, followed by 49.7 mg of DBU (0.32 mmol, 2 equiv.s). 998.9 mg of TGS (4.89 mmol, 30 equiv.s) were finally added and allowed to react for 6 hours. Volatiles were removed by centrifugal evaporation (Genevac EZ2 Elite) and the residue was dissolved in 45 mL of dichloromethane, filtered, and extracted against 5 mL of deionized water twice. The organic phase was dried over Na_2SO_4, filtered and concentrated again by centrifugal evaporation (Genevac EZ2 Elite). The polymerization kinetics was studied by monitoring the monomer consumption ^{1}H NMR (disappearance of monomer resonance at 2.86-3.02 ppm) in solutions of identical molarities in deuterated DMF. PTGS with nominal degree of polymerization = 60 was prepared in an identical fashion, using 60 equivalents of monomer and letting it react for 12 hours.

^{1}H NMR (DMSO); δ= 1.26 and 1.31 (ss, -O-C(C**H**$_3$)$_2$-O-), 2.86-3.02 (m, -[S-C**H**$_2$-C**H(**CH$_2$-R)-]S-), 3.44-3.65 (m, -[S-CH(-C**H**$_2$-O-R)-CH$_2$-]S- and 1 diastereotopic ***H*** of the CH$_2$ italicised CH(-O-C(CH$_3$)$_2$-O-C***H***$_2$-), 3.96-4.00 (s, 1 diastereotopic **H** of CH$_2$ -O-CH$_2$-CH(-O-C(CH$_3$)$_2$-

O-C***H*₂**-), 4.12-4.20 (q, -O-CH_2-C**H**(-O-C$(CH_3)_2$-O-CH_2), 7.19-7.22 (d, 2H, meta positions in phenyl group), 7.31-7.35 ppm (t, 3H, orto/para positions in phenyl group).

ATR FT-IR (thin film): 2986 (ν_{as} CH_3), 2929 (ν_{as} CH_2), 2872 (ν_s CH_2), 1461 (δ CH_2), 1365 (δ CH_3), 1110 (ν_{as} C-O-C), 1082 (ν_s C-O-C), 1049 cm^{-1} (ν_{as} C-O-C).

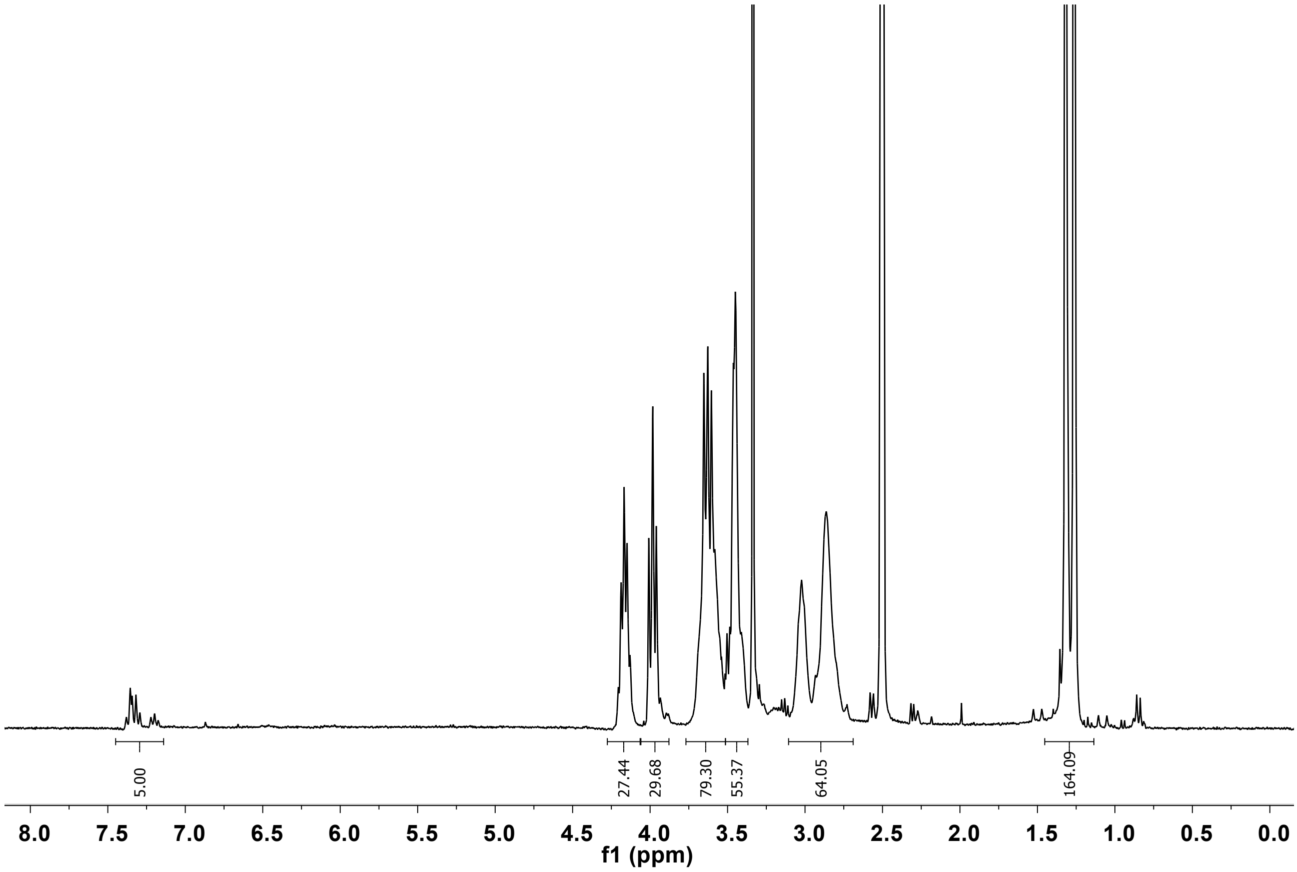

Figure S2. ^{1}H NMR of poly(thioglycidyl solketal)(PTGS) in DMSO-d_6.

<u>*Deprotection of PTGS to poly(thioglycidyl glycerol) (PTGG).*</u> 0.5 g of PTGS (0.1 mmol in sulfides) was dissolved into 3 mL of methanol containing 3 drops of water; the solution was transferred to a reaction flask containing 0.35 g of acidic ion exchange resin Dowex 50W X8 (70 wt. % of PTGS weight) activated with 1.0 M HCl prior to the reaction. The mixture was then heated to 50 °C and allowed to react for 24 hours without stirring. The resin was then removed by filtration and the volatiles were removed under reduced pressure to produce colorless viscous oil.

^{1}H NMR (D_2O); δ= 2.71-3.15 (m, -[S-C**H**$_2$-CH**(**CH_2-R)-]S-), 3.19-3.26 (s, -[S-CH_2-C**H(**CH_2-R)-]S-, 3.37-3.71 (m,-[S-CH-C**H**$_2$-O-C**H**$_2$-C**H**(-OH)-C**H**$_2$-OH) and (s, 1 diastereotopic **H** of CH_2 -O-CH_2-CH(-OH)-C***H*₂**-OH), 3.74-3.86 (s, 1 diastereotopic **H** of CH_2 (s, -O-CH_2-CH(-OH)-C***H*₂**-OH), 7.20-7.29 (d, 2H, meta positions in phenyl group), 7.32-7.47 ppm (t, 3H, orto/para positions in phenyl group).

ATR FT-IR (thin film): 3352 (ν OH), 2910 (ν_{as} CH_2), 2864 (ν_s CH_2), 1656 (ν C=O), 1101 (ν C-O-H secondary), 1032 (ν_{as} C-O-H primary)

Synthesis of PEG monomethyl ether thioacetate. The procedure is based on a Mitsunobu reaction adapted from a previous publication[1]. Briefly, 20.0 g (4 mmol of OH groups) of PEG monomethyl ether (5 kDa) were dissolved in 75 mL of dioxane. Under argon, the PEG solution was dried by refluxing the solution over a Soxhlet filled with activated 4 Å molecular sieves for 3 hours. In a separate flask, 2.10 g of PPh_3 (8 mmol, 2 equiv.s) were dissolved into 30 mL of previously dried dioxane and cooled to 0°C; 10 mL of dioxane containing 1.42 g of DIAD (7 mmol, 1.75 equiv.s) were then added dropwise and kept under magnetic stirring for 30 minutes. Using a double-ended needle, the dry PEG solution was added, stirring the mixture at room temperature for 30 minutes. A 50% dioxane/thioacetic acid (0.61 g of the latter; 7 mmol, 1.75 equiv.s) was finally added dropwise over 10 minutes. The mixture was allowed to stir for a further 1 hour, then heated at 50 °C and allowed to react overnight. Volatiles were removed under reduced pressure to provide viscous yellow oil. The oil was added to 120 mL of water and extracted against 30 mL of 1:1 ethyl acetate/diethyl ether (2x) then diethyl ether (2x); emulsions were broken by adding small amounts of ethanol. Sodium chloride (NaCl) was added to the water phase until the solution becomes cloudy, it was then extracted with 50 mL of dichloromethane (3x). The organic phases were then combined, dried over $MgSO_4$, filtered and concentrated *in vacuo*. The resulting oil was precipitated into diethyl ether, which was then placed in a -20 °C freezer for ~45 minutes, centrifuged and the liquid phase decanted; the pellets were dissolved in a minimum amount of dichloromethane and precipitated in an identical manner a further four times. The final white pellets were dried in a centrifugal evaporator (Genevac EZ2 Elite), followed by a vacuum oven at 45 °C for 24 hours resulting in 17.55 g of a waxy white solid (87.2% yield).
^{1}H NMR (400 MHz) ($CDCl_3$); δ = 2.32 (s, 3H, -CH_2-CH_2-S-C(=O)-C$\mathbf{H_3}$) 3.03–3.12 (t, 2H, -CH_2- C$\mathbf{H_2}$-S-C(=O)-CH_3), 3.37 (s, 3H, -CH_2-O-C$\mathbf{H_3}$), 3.52–3.56 (t, 2H, -C$\mathbf{H_2}$-O-CH_3), 3.57–3.72 ppm (t, -[(C$\mathbf{H_2}$)$_2$-O]-).

Synthesis of PTGG-phospholipid. 112 mg of PTGG (free thiol form; 0.0223 mmol, 1.2 eq.s) was dissolved into 2 mL of a 1:1 mixture of dichloromethane:methanol and mixed with 25 mg of 16:0 PE MCC (0.0268 mmol, 1 eq.) and allowed to stir overnight. The reaction mixture was concentrated then precipitated into diethyl ether (2x) from the 1:1 DCM:MeOH mixture.

Volatiles were removed *in vacuo* to give 111 mg of final product as a highly viscous colorless oil (81% yield).

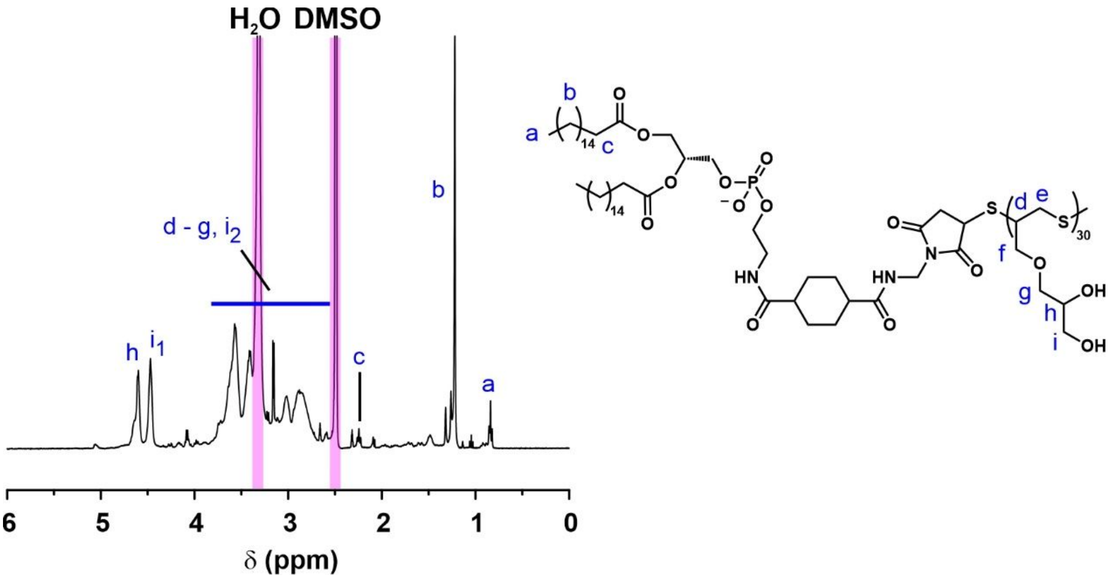

Figure S3. ^{1}H NMR spectra of PTGG-phospholipid in DMSO-d_6; letters correspond to the protons as assigned in the chemical structure.

<u>*Preparation of liposomes.*</u> Polymer-coated liposomes were prepared using a HEPC:Cholesterol:Polymer-lipid:DiD in a molar ratio of 61:33:5:1; 'naked' liposome controls had HEPC:Cholesterol:DiD in a molar ratio of 64:35:1. Briefly, all components were dissolved in chloroform or a chloroform-methanol mixture (only for PTGG-liposomes) and placed *in vacuo* to remove volatiles. The resulting thin film was rehydrated in 1 mL of sterile Hank's buffered saline (HBS) per 10 µmol of HEPC and the resulting dispersion was then extruded through a 200 nm filter membrane (x10) followed by a 100 nm filter membrane (11x). This process yielded PEGylated- and PTGGylated-liposomes of ~120 nm and 'naked' liposomes of ~100 nm in size (DLS).

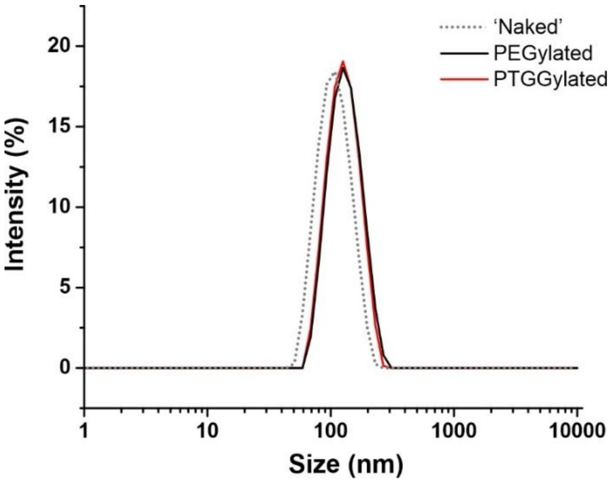

	Naked	PEGylated	PTGGylated
Z-Average (nm)	102	124	121
PDI	0.072	0.070	0.052

Figure S4. *Left*: DLS intensity-based size traces of the naked, PEGylated or PTGGylated liposomes. *Right*: table of the calculated Z-average and PDI

The resulting liposomes were diluted with a 1 M trehalose-HBS to form a solution with a 10:1 mole ratio of trehalose:lipid. These were then aliquoted, snap-frozen in liquid nitrogen and stored in a -80˚C freezer until further use.

S1.3.3. Assessment of complement activation.

A C3a and C5a sandwich ELISA was performed using Platinum (eBioscience, San Diego) ELISA kit to measure complement activation in human plasma after incubation with PTGG, PEG or zymosan (positive control) substrates or sterile (endotoxin free) PBS (negative control). For measurement of complement activation, the reaction was started by adding 0.8, 0.4 or 0.1 mg/mL of substrate to 1X undiluted human plasma (150 µL in sterile Eppendorf tubes, n = 3) and kept in a water bath at 37°C for 30 min. The complement cascade was stopped by diluting serum with the kit provided inhibitor. Soluble activation products (C3a and C5a) remaining in the serum were quantified by sandwich ELISA targeting the soluble neo-antigens of cleaved complement proteins C3 and C5 using respectively the human C3a or C5a Platinum ELISA kits according to the manufacturer's protocols. Briefly, to each well of the plate (pre-coated with a target specific capture antibody) was added 100 µL of either C3a/C5a standard or sample, covered with a plate sealer, and incubated for 1 hr at 37°C. The liquid of each well was aspirated and 100 µL of (1x) biotinylated detection antibody was added to each well, covered with a plate sealer, and incubated for 1 hr at 37°C with gentle agitation. The plate was then washed by adding 350 µL of wash buffer and allowing ~120 seconds before aspirating the liquid; this wash step was repeated for a total of three washes. 100 µL of 1x HRP-Conjugate working solution was then added to each well, covered with a new plate sealer, and incubated for 30 minutes at 37°C. The solution was aspirated from each well and washed 5x with wash buffer (*vide supra*). 90 µL of kit provided TMB substrate solution was then added to each well, covered with a new plate sealer, and incubated for ~15 minutes at 37°C where optimal color development was achieved; 50 µL of kit-provided stop solution were then added to each well and the absorbance was measured at 450 nm using a microplate reader.

S1.3.4. Preparation of protein conjugates

<u>*A) Lysozyme conjugates.*</u> 5 or 10 kDa PTGG (75 µmol, totaling 375 or 750 mg for the 5 and 10 kDa PTGG respectively) solutions in a mixture of DMF/degassed water (600 µL/4.5 mL, respectively) were introduced into the reaction vessels of a 12-position Carousel parallel reactor (previously purged with argon for 10 mins), followed by the addition of 150 µL of

aqueous 1 M $NaBH_4$ (2 equiv.s per thiol). The mixture was allowed to stir for 30 minutes, then excess $NaBH_4$ was decomposed by acidifying the mixture to pH 4.5 with few drops of acetic acid and allowing to stir for further 20 minutes. After adjusting the pH to 7.4 with a 1 M NaOH solution, a solution of 20.0 mg of BMPS (75 µmol) in 150 µL of DMF was added. 36.0 mg of lysozyme (2.55 µmol) dissolved into 1.5 mL of pH 7.4 PBS were then added 2 minutes after BMPS. This corresponds to a 5:1 molar ratio between PTGG chains and lysozyme lysines. The solutions were stirred for 5 hours under argon at 25 °C, after which residual NHS-esters were quenched by adding Tris base (9.2 mg, 75 µmol), raising the pH to 8, allowing 15 minutes to react. The conjugates were then immediately purified via ultrafiltration (10 mL Amicon vessel) using a 10 kDa MWCO membrane followed by dialysis using a 20 kDa MWCO (overnight) for the 5 kDa polymer-lysozyme conjugates, or ultrafiltrated using a 30 kDa MWCO for the 10 kDa PTGG conjugate.

PEG-lysozyme conjugates were synthesized with an identical method (5 or 2.5 equiv.s of polymer per lysine) using PEG (5 kDa)-thioacetate in place of PTGG. Please note: the basification caused by $NaBH_4$ is sufficient to deprotect the thioacetate into a thiol/thiolate and acetate.

B) Ovalbumin (OVA) conjugates (rat studies). 10 mg of OVA were dissolved in 0.75 mL of 20 mM POPSO buffer and brought to pH 8.1 using 1 M sodium bicarbonate. Separately, 5 kDa PTGG (150 mg, 30 µmol, 15 mg of PTGG/mg protein) in a mixture of isopropanol/degassed water (100 µL and 1.75 mL, respectively) were introduced into a separate reaction vessels of a 12-position Radley's carousel parallel reactor (previously purged with argon for 10 mins), followed by the addition of 15 µL of aqueous 1 M $NaBH_4$. The mixture was allowed to stir for 30 minutes, then remaining $NaBH_4$ was decomposed by acidifying the mixture to pH 4.5 with few drops of glacial acetic acid followed by 1 M HCl. After adjusting the pH to 7.4 with 1 M sodium bicarbonate solution, 7.98 mg of BMPS (30 µmol) in 100 µL of dry DMF was added. After 30 seconds, the PTGG solution was mixed with the OVA solution and the pH was confirmed to be between 8-8.2 or adjusted with 1 M sodium bicarbonate if required. After 1.5 hour of reaction 0.5 mg of FITC dissolved in 0.5 mL of dry DMF was slowly added. The solutions were stirred for a further 5 hours at 25 °C, after which residual NHS-esters and FITC were quenched by adding Tris base (12.2 mg, 100 µmol, in 1 mL). The conjugates were then immediately purified via dialysis using a 50 kDa MWCO membrane.

PEG-OVA conjugates were synthesized in an identical method except for using PEG(5 kDa)-thioacetate in place of PTGG at 7.5 mg of PEG/mg protein (75 mg in total for 10 mg protein, 15 umol, 3.25 mg of BMPS).

C) Ovalbumin (OVA) conjugates (mice studies). To 150 mg of ovalbumin dissolved in 15 mL of pH 6.5 phosphate buffered water (0.1 M phosphate) was added 5 mg of Sulfo-Cy5-maleimide in 1 mL of DMF (added drop wise). The reaction vessel was protected from light and allowed to react for 24 hours. The solution was dialyzed in a 30 kDa MWCO membrane against PBS followed by deionized water then freeze-dried to give 135 mg of Sulfo-Cy5-labelled OVA. Polymer-OVA conjugates were synthesized in an identical procedure as for the polymer-OVA conjugates in the rat studies however Sulfo-Cy5-labelled OVA was used in place of pristine OVA and no FITC was used.

S1.3.5. Characterization of protein conjugates

A) SDS-PAGE. Electrophoresis was carried out using a 4-20% Mini-PROTEAN® TGX™ Precast Protein Gels (Biorad, Hercules, USA). Briefly, 15 µL of a 20 µg protein/mL solution of lysozyme or lysozyme conjugate was added to 15 µL of 2x Laemmli sample buffer, heated to 90 °C for 5 min then centrifuged at 300 rpm. Samples were loaded in each lane and allowed to run at 60 volts for 10 min, and then at 200 volts for 35 min until the dye front reached the bottom of the gel. Gels were fixed by washing in 100 mL of 40% ethanol/10% acetic acid for 15 mins, then washed in water and stained with Coomassie blue.
B) BCA assay. A microplate assay was performed by mixing 20 µL of protein-containing solution (for example, from freeze-dried protein, a 1 mg/mL PTGG30/Lys solution in PBS) and 200 µL of BCA working reagent as per the supplier's protocol. The working solution is prepared by mixing 100 parts of reagent A (containing bicinchoninic acid) with two parts reagent B (containing 4% (w/v) copper (II) sulfate pentahydrate) to form a green solution. Protein standard curves (using bovine serum albumin (BSA)) were obtained via serial dilution of an original 1 mg/mL solution. Each standard and protein samples were incubated at 37 °C for 30 min to allow the sample to equilibrate to room temperature before reading. Microplates were covered during incubation to avoid evaporation of the sample. Absorbance at 562 nm was then measured and a standard curve was prepared by plotting the measurement for each BSA standard vs. its concentration in µg/mL. The standard curve was then used to determine the protein concentration of the protein-conjugate samples. The discrepancy between weighed conjugate concentration and calculated protein concentration

from BSA assay was determined to be the polymeric weight fractions. Number of chains per protein were calculated as: $\left(\frac{Mw\ of\ protein}{\left(\frac{[conjugate]}{[protein]}\right)} - Mw\ of\ protein\right) \div Mn\ of\ polymer$

C) Fluorescamine assay (OVA conjugates only). 40 uL of PBS solutions of ethanolamine, OVA or its conjugates were dispensed in individual wells of a black half area 96-well plate followed by the addition of 12 μL of a 3 mg/mL fluorescamine solution in DMSO. Fluorescence was measured using a BioTek microplate reader at λ_{ex} = 365 nm and λ_{em}= 470 nm.

The available amines per OVA were quantified by using a dilution series, first obtaining a molar amine concentration in each sample through the use of a calibration with ethanolamine, and then relating the results to the OVA molar concentration (obtained from its concentration in mg/mL divided by the 'monomeric' molecular weight obtained via AF4). The residual amines present in OVA after its conjugation with PEG or PTGG chains were quantified by measuring in triplicate stock solutions of the two conjugates (resp. 2.50 and 2.26 mg/mL in protein, as determined using Pierce BCA assay kit according to manufacturer's instructions), and then relating the data to the fluorescence emission of OVA solutions of similar protein concentration (Figure S4A).

D) Enzymatic activity (lysozyme conjugates only). The activities of lysozyme and lysozyme-PTGG_conjugates were determined using the EnzChek® Lysozyme Assay Kit (Thermo Fisher) which measures the lytic activity toward dye-quenched *Micrococcus lysodeikticus* cell wall. Following the manufacture's protocol, 100 μL of lysozyme/lysozyme conjugate solution (0.5 mg/mL of protein content) was added to a black 96-well plate and mixed with 100 μL of lysozyme substrate (50 μg/mL). The reaction mixtures were then incubated for 60 min at 37 °C taking a reading every 10 minutes (excitation 485 ± 20 nm; emission at 528 ± 20 nm). The background fluorescence was corrected by subtracting the value derived from the no-enzyme controls.

E) ELISA assays. The recognition of lysozyme and lysozyme-conjugates by the anti-lysozyme antibody was assessed using an enzyme-linked immunosorbent assay (ELISA). High protein binding ELISA plates were incubated with lysozyme or lysozyme conjugates (200 ng of protein per well) at 4°C in 0.01 M PBS, pH 7.4. After 15 hours the solution was removed, and the protein content of the removed solution was measured using a high sensitivity BCA assay kit in order to normalize for differences in lysozyme binding (see section B). Plates were washed with PBS and blocked by incubation with 1% (w/v) bovine serum albumin (BSA) in PBS for 1 hr at room temperature and finally washed with PBS/Tween (PBS containing 0.05% Tween 80, pH 7.6).

Plates were then incubated with 100 uL of a 0.1 mg/mL solution of either monoclonal human anti-chicken hen egg lysozyme antibody (clone AbD11397, 0.1 mg dissolved in 1 mL of 1% Tween 80 in PBS) or a polyclonal rabbit anti-chicken lysozyme antibody (ab34799, ABCAM, sold as 80 mg/mL solution). After incubation for 1 hr at room temperature, plates were washed with PBS/Tween 80 and incubated with a HRP-goat anti-human IgG antibody (diluted 1:5000 in PBS/Tween 80)(62-8420, Thermo Fisher) was used as the secondary antibody for the monoclonal human Ab and or a HRP-anti-rabbit IgG (7074, Cell Signaling Technology, Massachusetts, USA) as secondary antibody for the monoclonal human Ab and for the rabbit polyclonal anti-lysozyme antibody, respectively, and allowed 1 hr at room temperature to bind. The plates were first washed 5 times with 1% Tween 80 in PBS then 100 µL of TMB ELISA Substrate (High Sensitivity) solution was added to each well and allowed to incubate for ~15 mins in the dark, before addition of stop solution (50 µL of 2 M H_2SO_4). The absorbance at 450 nm was recorded using a microplate reader and results were normalized to the initial binding of free lysozyme on the plate (as determined by the BCA assay) (absorbance of lysozyme-conjugate÷ [mass of lysozyme conjugate bound÷mass of unmodified lysozyme bound])÷ELISA absorbance of unmodified lysozyme x 100). Note: the binding of lysozyme conjugates was extremely low compared to free lysozyme; therefore serial dilutions of lysozyme were screened and a concentration with similar degree of binding as the conjugate was used for better comparison.

F) Stability upon lyophilization (freeze drying). Aliquots of each lysozyme or lysozyme conjugate sample were frozen by immersion in liquid nitrogen before H_2O removal via freeze-drying. The samples were lyophilized for 12 hr per cycle at a pressure under 1 mbar. After each cycle, deionized milliQ H_2O was added to redissolve the lysozyme or lysozyme-conjugate powder at a protein concentration of 1 mg/mL, before being refrozen and freeze-dried for additional cycles. A 50 µL sample was taken on the 1st, 5th, 8th and 10th cycle, mixed with 50 µL of 2X PBS and measured for activity using the EnzChek® Lysozyme Assay Kit (Thermo Fisher) as above described in section D.

G) Asymmetric Flow Field-Flow Fractionation (AF4) was performed as described in section S1.2 on 2.5 mg/mL (protein) solutions of OVA or its conjugates.

S1.3.6. Evaluation of lysozyme (conjugate) degradability

A) Proteolytic degradation. The proteolytic enzymes pepsin, trypsin, chymotrypsin, carboxypeptidase Y, and aminopeptidase were used to evaluate the susceptibility to proteolysis. Native lysozyme or PTGG30/Lys were dissolved at a concentration of 2 mg protein/mL (as determined via BCA assay and possibly adjusted) in 0.5 mL of PBS and mixed with 0.5 mL of solutions of given proteases in the same buffers to achieve final concentration of 1 mg of protein/mL of lysozyme or lysozyme-conjugate and either 10 mg/mL (for trypsin, pepsin and chymotrypsin) or 1 mg/mL (for carboxypeptidase and aminopeptidase) of peptidase. The solutions were incubated at 37 °C for 24h, sampling 200 μL aliquots were taken after 24 hr incubation and measured for activity (see above).

B) Oxidative damage. All oxidation reactions were performed in glass reactor vessels by mixing a stock solution of native lysozyme or lysozyme-PTGG conjugate at 37 °C. All the reactions were quenched by adding 1 mL of a 35 μg/mL sodium ascorbate solution and the residual enzymatic activity was evaluated using the EnzChek® Lysozyme Assay Kit as described above.

Hydrogen peroxide (H_2O_2). 982 μL of a 1.02 mg of protein/mL solution of lysozyme or lysozyme-conjugate was mixed with 18 μL of 3.27, 1.63, 0.98, 0.65, 0.33 or 0.16 M aqueous H_2O_2 (respectively, this corresponds to a 10:1, 5:1, 3:1, 2:1, 1:1 or 0.5:1 H_2O_2:thioether stoichiometric ratio in the lysozyme-PTGG conjugate) and incubated for 24 hrs before quenching (as described, *vide supra)*. Note: moles of thioether in PTGG30/Lys was calculated based on the measured number of PTGG chains per lysozyme in Table 2 (2.5 chains) and 31 thioethers per PTGG chain (initiator S + 30 monomeric S) which was determined to be ≈ 5.4 μmoles/mg of lysozyme.

Superoxide ($O_2^{\cdot -}$). 500 μL of a 2 mg of protein/mL solution of lysozyme or lysozyme-conjugate was mixed with 500 μL of a freshly prepared 107.8, 53.9, 32.3, 21.6, 10.8 or 5.4 μM aqueous KO_2 solution (respectively, this corresponds to a 10:1, 5:1, 3:1, 2:1, 1:1 or 0.5:1 H_2O_2:thioether stoichiometric ratio in the lysozyme-PTGG conjugate) and incubated for 3 hrs before quenching *ut supra*.

Hypochlorite (ClO^-). Respectively, 910, 955, 973, 982, 991 and 996 μL of a 1.02 mg of protein/mL solution of a lysozyme or lysozyme-conjugate was mixed with 36, 18, 10.8, 7.2, 3.6 or 1.8 μL of a 1.5 M sodium hypochlorite solution (corresponding to a 10:1, 5:1, 3:1, 2:1, 1:1 or 0.5:1 H_2O_2:thioether stoichiometric ratio in the lysozyme-PTGG conjugate) and were incubated for 15 minutes before quenching (*ut supra*).

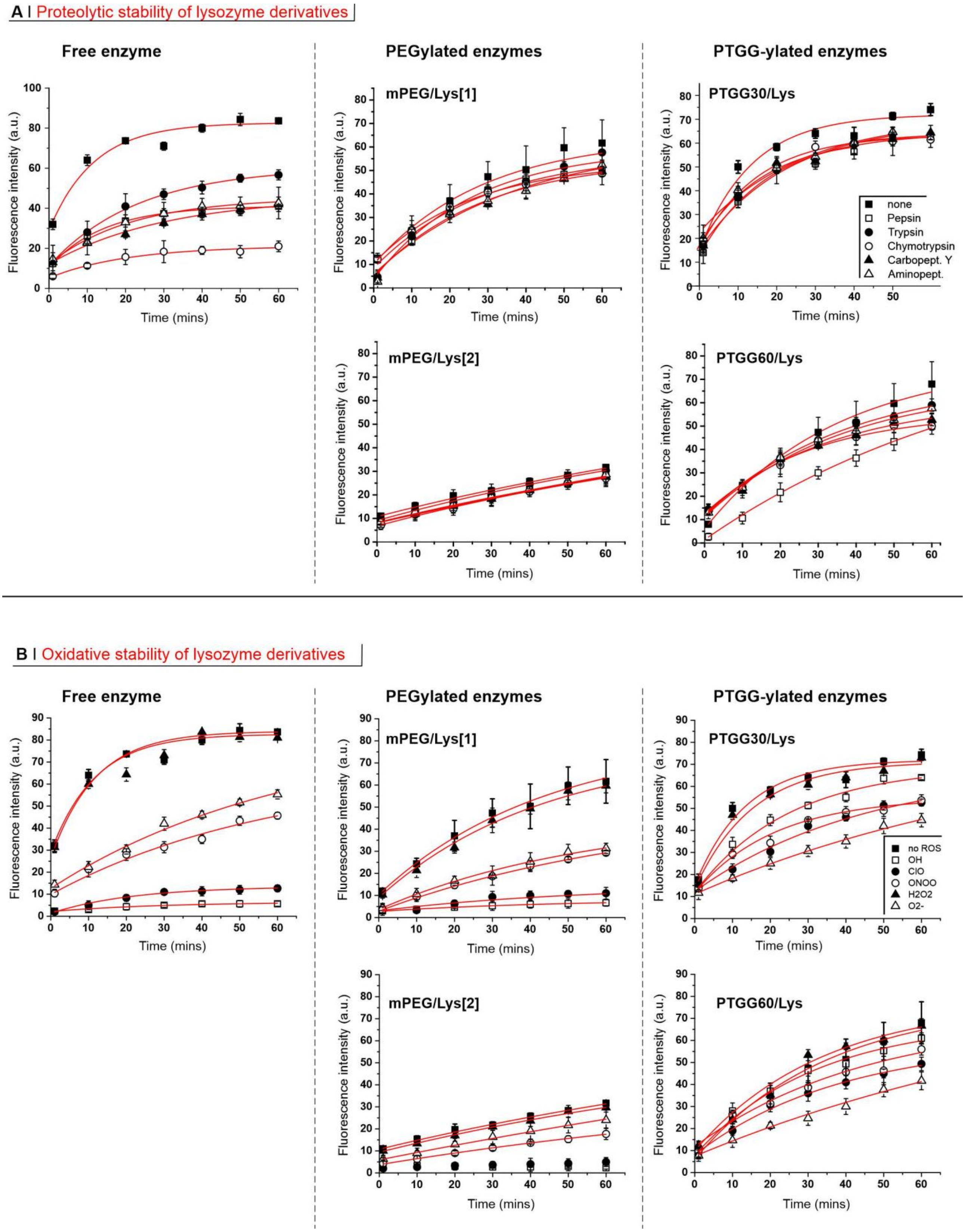

Figure S5. Lysozyme activity was assessed using a dye-quenched assay, keeping the protein concentration constant at 0.5 mg/mL; the fluorescence was monitored over 60 minutes at 37ºC, fitting the data with an exponential growth equation fluorescence = A1*exp(time/τ) + A2 (inset; red lines are fittings). A rate constant is calculated as 1/τ, while the sum A1+A2 provides the fluorescence at plateau.

Peroxynitrite ($ONOO^{\cdot -}$). 500 μL of a 2 mg of protein/mL solution of lysozyme or lysozyme-conjugate was mixed with 500 μL of freshly prepared 107, 53.7, 32.2, 21.5, 10.7 or 5.4 μM aqueous SIN-1 chloride solution (respectively, this corresponds to a 10:1, 5:1, 3:1, 2:1, 1:1 or 0.5:1 H_2O_2:thioether stoichiometric ratio in the lysozyme-PTGG conjugate) and incubated for 3 hrs before quenching.

Hydroxyl radical (·OH). 880, 940, 964 976, 988 or 994 μL of aqueous $CuCl_2$ were prepared respectively at 6.11, 2.86, 1.67, 1.10, 0.54 or 0.27 μM. Correspondingly, these solutions were mixed with 120, 60, 36, 24, 12 or 6 μL of a 0.898 M of H_2O_2 (1:10 Cu:H_2O_2 mole ratio); 0.5 mL of the $CuCl_2$/H_2O_2 mixture was then immediately added to 0.5 mL of a solution containing 2 mg of protein/mL of lysozyme or lysozyme-PTGG conjugate (respectively, this corresponds to a 10:1, 5:1, 3:1, 2:1, 1:1 or 0.5:1 H_2O_2:thioether stoichiometric ratio in the lysozyme-PTGG conjugate). The mixture was incubated at 37ºC for 24 hrs before being quenched with ascorbic acid (*ut supra*).

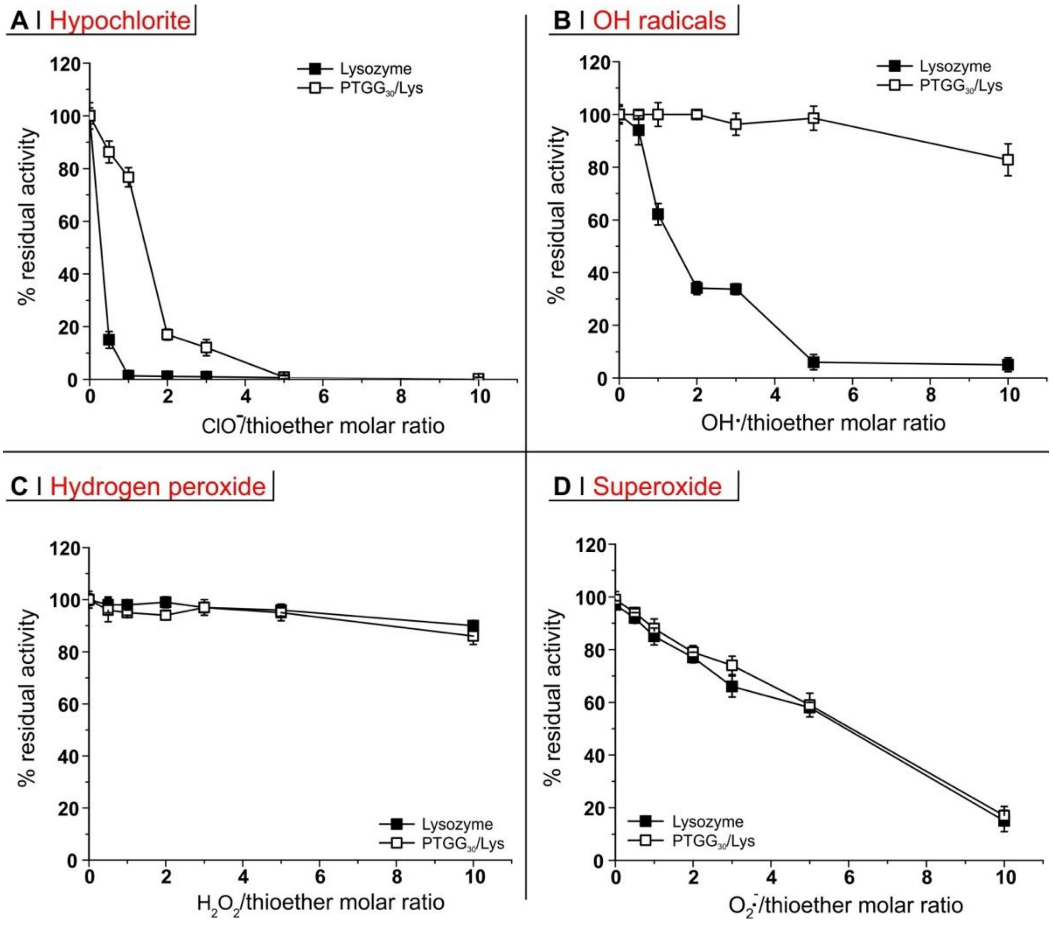

Figure S6. Residual activity (expressed as the % of the fluorescence at plateau in the absence of ROS) as a function of the molar ratio between the various ROS and the thioethers in the PTGG chains. The lysozyme controls had the same lysozyme concentration as the experiments with the conjugates.

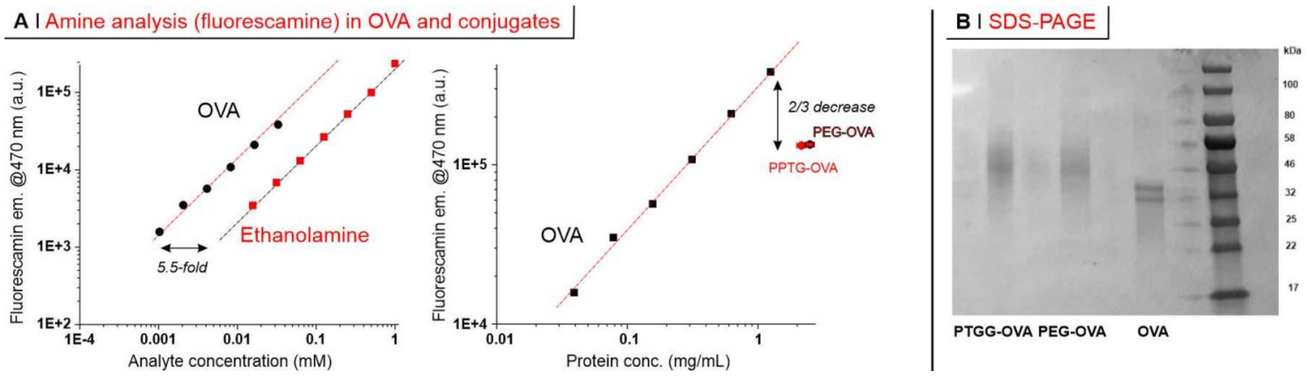

Figure S7. ***A.*** *Left.* Results of the fluorescamine assay on OVA, compared to a monofunctional, low MW amine (ethanolamine), showing an average number of 5.5 free amines per OVA molecule. *Right.* The conjugates show a 2/3 decrease in fluorescence in comparison to free OVA at the same protein concentration, which would to conjugation corresponds to an average of 3.3 chains for PTGG, 3.6 for PEG. ***B.*** SDS-PAGE gel of OVA, PTGG-OVA and PEG-OVA.

Table S1. Molecular weight characterization of OVA and its conjugates.

Sample	Amine analysis	AF4 analysis [c]						SDS-PAGE
		1st peak			2nd peak			
	$\overline{M_n}$ (kDa) [a] / *Chains per OVA*	$\overline{M_n}$ (kDa)	Đ	*%wt.*	$\overline{M_n}$ (kDa)	Đ	*%wt.*	*Chains per OVA*
OVA	=	34.3	1.01	67%	70.1	1.02	33%	=
PTGG-OVA	50.8 / *3.3*	38.9	~1	63%	56.4	1.07	37%	*2-3*
PEG-OVA	52.5 / *3.6*	45.5	1.10	48%	58.3	1.13	52%	*2-3*

[a] Values calculated using the OVA M_n (in its monomeric form) from AF4, added of the number of chains obtained via the fluorescamine assay multiplied by 5kDa (molecular weight of the PEG or PPTG chains).
[b] From the fluorescamine assay.
[c] Determined via A4F, fitting the traces with a multiple Gaussian peak model. Please note that OVA-PEG actually presents a broad ‘tail’ at high MW values, which lends for a fitting with any number of additional peaks; we here report data for a two-peak fitting only.

S2 Cell culture

A) General Procedures. HDFn and RAW 264.7 cells were cultured in DMEM supplemented with 10% FBS, 1% antibiotics, 2 mM glutamine, 50 µg/mL ascorbic acid and 10 mM BGP. All cells were cultured at 37 °C under 5% CO2 and used to maximum passage number of 15.

B) Cell Viability (MTS). RAW 264.7 were detached by scraping whereas HDFn were trypsinized and seeded in a 96-well plate (10,000 cells per well) and allowed to adhere overnight. Cells were washed the next day with PBS and 100 µL of fresh medium, then 100 µL of PEG or PTGG solutions in DMEM (2X media was used for dilutions) at concentrations of 0.01,0.05, 0.1, 0.5, 1, 5 or 10 mg mL^{-1} were added; 5% DMSO was used as a positive control. After 24 or 48 hrs incubation, the medium was discarded; cells were gently washed with PBS and incubated for 1 h at 37 °C with medium containing Cell Titer 96 Aqueous One Solution Reagent as per the manufacturer's instructions. Formazan absorbance was measured at 490 nm and results are expressed as percentage of the untreated control cells.

C) Assessment of anti-oxidant activity (H_2O_2 / ClO^- scavenging). The production of reactive oxygen species (ROS) (H_2O_2/ClO^-) in stimulated RAW 264.7 macrophages was assessed using the Hydrogen Peroxide (absorbance at 535 nm) and Hypochlorite Assay Kits (absorbance at 555 nm), respectively. Both kits were used in accordance with manufacturer's instructions. This was carried out by platting the RAW 264.7 macrophages in DMEM complete medium in transparent 96-well flat-bottomed plates at a concentration of 2.5×10^4 cells per well at 37 °C in an incubator under a humidified atmosphere of 5% CO_2: 95% air. After 24 h the cells were washed with PBS and treated with PTGG/PEG conjugates at concentrations of 0.1, 0.5, 1, 2, and 4 mg/mL in DMEM complete medium under stimulating condition (LPS at concentration of 500 ng/mL).

D) Assessment of anti-inflammatory effects. RAW 264.7 macrophages were plated in 24-well flat-bottomed plates at a density of 1×10^5 cells per well in DMEM complete medium and incubated at 37°C and 5% CO_2. Cells were washed the next day with PBS and 500 µL of a solution containing 500 ng/mL LPS alone or with PTGG/PEG conjugates at concentrations of 0.01, 0.05, 0.1, 0.5, 1, 5 or 10 mg mL^{-1} were added into each well. After 24 h of incubation, the supernatants were collected and centrifuged for 5 minutes at 13,000 rpm. TNF-α concentrations were determined with the Cohesion Bioscience Mouse TNF-α ELISA (Enzyme-Linked Immunosorbent Assay) kit. Briefly, capture antibody solution was added to the wells of a 96-well plate and allowed to coat the surface overnight. Plates were blocked with 1% BSA in PBS for 1 h at room temperature, then washed in PBS and 0.1%

Tween 80 prior to the addition of 100 μL of supernatant from stimulated RAW246.7 macrophages. After 1 h incubation, wells were washed and incubated with biotinylated anti-TNFα antibody for an additional 1 hr. After washing, streptavidin-conjugated HRP was added and allowed 30 minutes to bind. The plates were washed with wash buffer again and the reaction was allowed to develop for 10–30 min in the presence of TMB substrate. After addition of the stop solution absorbance was measured at 450 nm.

E) Uptake in LPS-activated macrophages.

E1) Polymer labelling with fluorescein (FITC – fluorescein isothiocyanate). 100 mg of 5kDa PTGG-SH or PEG-thioacetate (0.02 mmol) were dissolved in 1 mL of degassed deionized water and introduced into the reaction vessels of a 12-position Carousel parallel reactor (previously purged with argon for 10 mins), followed by the addition of 40 μL of aqueous 1 M $NaBH_4$ (2 equiv.s per thiol or thioacetate). The mixture was allowed to stir for 30 minutes, and then excess $NaBH_4$ was decomposed by acidifying the mixture to pH 4.5 with few drops of acetic acid and allowing to stir for further 20 minutes. After adjusting the pH to 7.4 with a 0.1 M NaOH solution, a solution of 8 mg (0.02 mmol) of FITC in 100 μL of DMF was added. The solutions were stirred overnight under argon at 25 °C, then dialysed against deionized water for 3 days, until free dye molecules are completely removed.

E2) Uptake in activated macrophages. RAW 264.7 macrophages were plated in black-walled flat-bottom 96-well plates at a density of 2.5 × 10^4 cells per well and incubated in DMEM complete medium at 37 ◦C in a 5% CO_2 atmosphere overnight. Cells were washed with PBS then incubated for 24 h with LPS at concentration of 500 ng/mL then subsequently exposed to LPS again at 500 ng/mL together with FITC-labelled PTGG/PEG conjugates at 0.1, 0.5, 1, and 2 mg/mL in 100 μL of complete medium and incubated for a further 24 hours. After washing the cells 3x with PBS, cells were lysed with a 0.5% solution of Triton X-100 in 0.2 N NaOH for 10 minutes at 37◦C. The fluorescence of cell lysates was measured in a plate reader using an 485 ± 20 nm excitation fileter and recording the emission with a 528 ± 20 nm filter. Polymer concentrations were determined from standard curves of FITC-labelled polymer in untreated cell lysates.

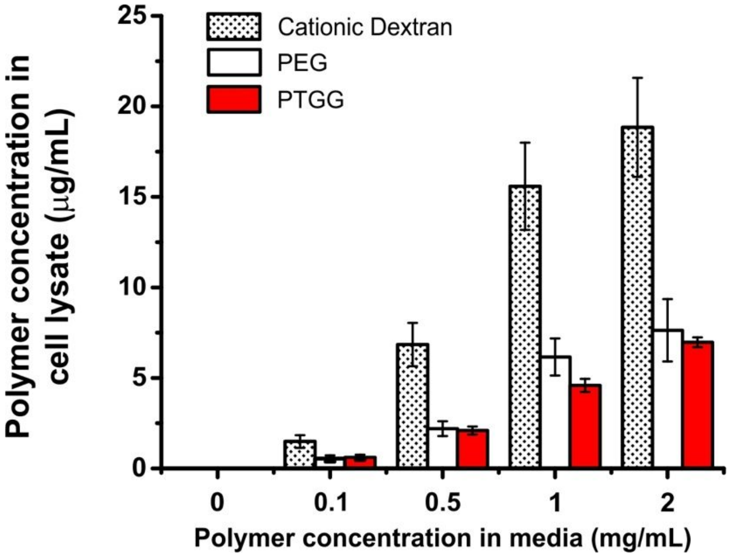

Figure S8. Uptake of FITC-labelled polymers into RAW 246.7 macrophages after 24 exposure (fluorescence measured in cell lysates, n=3)

S3. *In vivo* studies

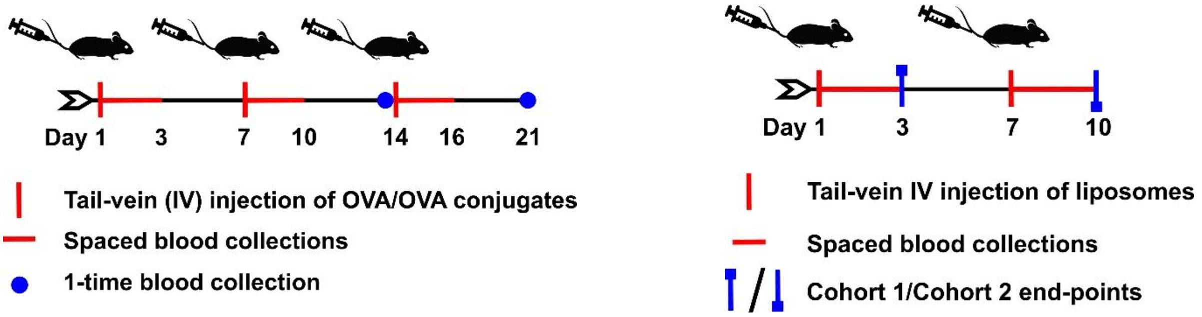

Scheme S1. Timeline of the experiments in mice for OVA and its conjugates (left) and for liposomal formulations.

Pharmacokinetic study of OVA conjugates and immunoglobulin profiling in mice.

Female CD-1 (26-30 day [~ 4 week] old) mice were purchased from Charles River and allowed to acclimatize for 2 weeks before initiation of experiments. Mice were treated with either OVA(-Sulfo-Cy5), PEG/OVA(-Sulfo-Cy5) or PTGG/OVA(-Sulfo-Cy5) at 500 µg of protein/kg at day 1, 7 and 14; 100 µL of sterile saline was injected at day 1, 7 and 14 as a control group. Mice were tail-nicked and ~5 µL of blood was collected in heparinized capillary tubes (Kimble) at 0.017, 0.25, 0.75, 1.5, 4.5, 24, 48 and 72 hours post-injection. Collected blood was diluted into 0.1% Tween 20 and analyzed on a plate-reader spectrofluorimetry (excitation 640 nm, emission 690 nm); data were fitted to a two-compartment bolus model in PKSolver to determine pharmacokinetic parameters (n=5). Treatment groups at each dose were analyzed for statistical significance using a two-way ANOVA with a Sidak's multiple comparison analysis.

Note: upon third injection of either OVA or PEG/OVA, a severe anaphylactic response was seen in 3/5 of OVA-treated mice resulting in 2 deaths, and severe anaphylaxis in 2/5 of PEG/OVA-treated mice (no deaths). No such response was observed in the PTGG/OVA-treated mice.

Antigenicity experiments. For each treatment group, one cohort of mice was sacrificed at day 14 (2 injections) and a second cohort at day 21 (3 injections); 1 week after their final injection, blood was collected in an EDTA-coated syringe, and serum was separated from blood cells by centrifugation at 1500 g for 5 minutes. Serum from each mouse was aliquoted, immediately frozen in liquid nitrogen and stored at -80 °C until further use.

Anti-OVA levels (ELISA). 500 ng/well of OVA in 100 µL of solution were incubated overnight in Nunc MaxiSorb plates in pH 10.5, 50 mM carbonate buffer at 4 °C. The next day, wells

were washed 3 times with wash buffer (0.05% CHAPS in pH 7.4 Tris buffered saline (TBS)). Plates were then blocked with 3% BSA in TBS (OVA blocking buffer) for 5 hours at room temperature. 100 µL of serially diluted serum in blocking buffer with dilution ratios of 1:25, 1:50, 1:100, 1:200, 1:400, 1:800, 1:1600, 1:3200, 1:6400, 1:12800 and 1:25600 were added to each well and allowed to incubate for 2 hours. The plates were washed 5x in wash buffer then 100 µL of 80 ng/mL (10,000x dilution) of HRP-conjugated goat anti-mouse IgM (EMD Millipore, cat no. AP128P) or rabbit anti-mouse IgG (EMD Millipore, cat no. AP160P) in blocking buffer were added to each well and incubated for 1 hour. Plates were washed a further 5x in wash buffer before 100 µL/well of HRP substrate, TMB/H_2O_2, was added and allowed to react for 15 minutes before being stopped with 100 µL of 2 M H_2SO_4. Absorbance was then read at 450 nm and each value was subtracted by a blank (no serum) control. Anti-OVA IgM and IgG antibody titers were quantified as their reciprocal endpoint titers with a 95% confidence interval in accordance with reference[2].

Anti-polymer levels (ELISA). 50 µL of either PTGG-phospholipid or 16:0 PEG5000 PE in ethanol at 400 nmol/mL was added to each well and allowed to air-dry over 5 hours on Immulon 1B (hydrophobically surface-treated) 96-well plates (ThermoFisher, Cat. No: 14-245-78). The wells were washed 3x with wash buffer (0.05% CHAPS in TBS) and blocked with 0.5% casein in PBS (anti-polymer blocking buffer) for 3 hours. Serum was serially diluted into anti-polymer blocking buffer at 1:25, 1:50, 1:100, 1:200, 1:400, 1:800, 1:1600, 1:3200, 1:6400, 1:12800 and 1:25600 and added at 100 µL/well for 2 hours. Plates were washed a further 5x in wash buffer and 100 µL of either HRP-conjugated goat anti-mouse IgM or rabbit anti-mouse IgG was added (80 ng/mL) and allowed to incubate for 1 hour. Wells were washed a further 5x in wash buffer, then exposed to 100 µL of TMB/H2O2 substrate for 15 minutes before being stopped with 100 µL of 2 M H_2SO_4. Absorbance was then read at 450 (signal) and a blank (no serum) control was subtracted from each value. Anti-PEG and anti-PTGG IgM and IgG antibody titers were quantified as their reciprocal endpoint titers with a 95% confidence interval in accordance with reference[2]. Titers of less than 25, the lowest dilution evaluated, were reported as 25.

Note: CHAPS was used instead of PEGylated surfactants (e.g. Tweens) which can interfere with this assay/experiment.

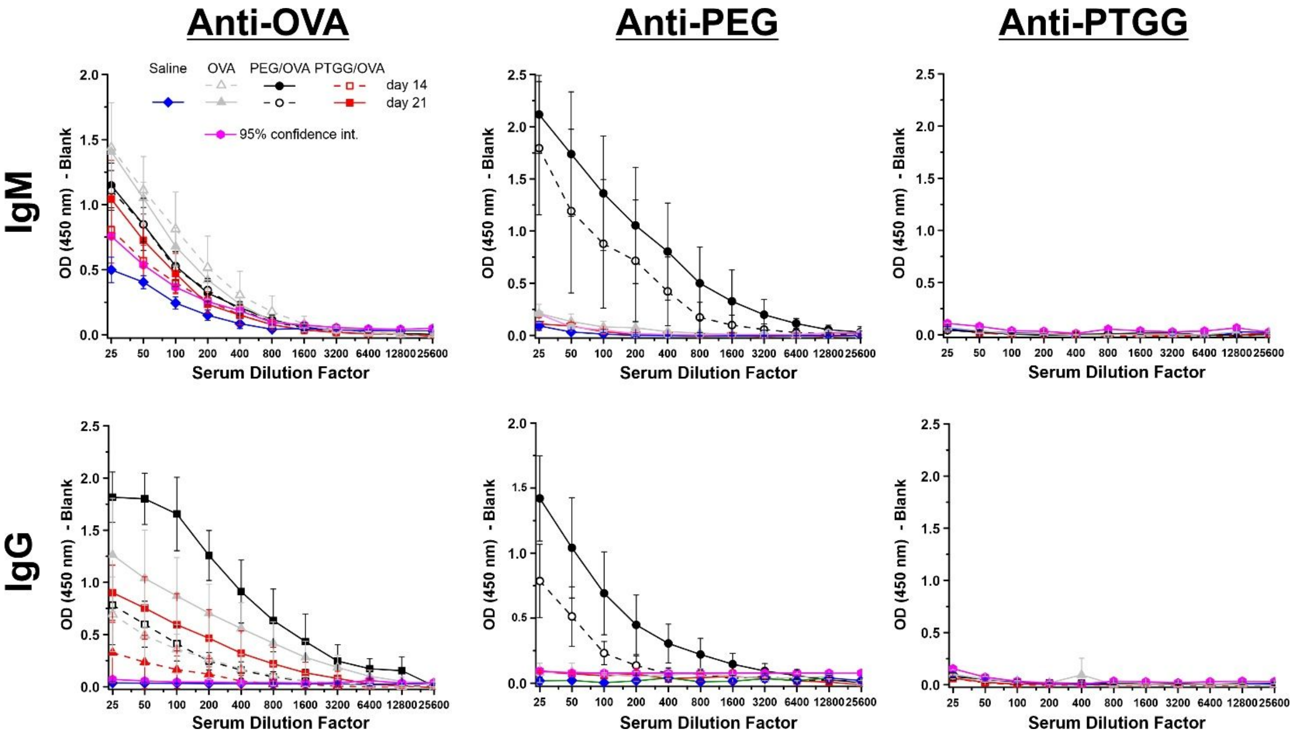

Figure S9. Evaluation of reciprocal endpoint for both IgM (top row) and IgG (bottom row) antibodies via direct ELISA with either OVA (left panel), PEG (center panel) or PTGG (right panel) antigen coated plates. (*n* = 3-5)

<u>*Pharmacokinetic study and biodistribution of liposomes (mice).*</u> Male BALB/c mice (4-5 weeks old) were purchased from Charles River and allowed to acclimatize for 2 weeks before initiation of experiments. Mice were split in to two cohorts; those receiving either 1 dose or 2 doses of liposomes. Those receiving 1 dose received 10 μmol of lipid/kg of either 'naked' liposomes, PEGylated liposomes or PTGGylated liposomes on day 1; those receiving 2 doses received a first 0.1 μmol of lipid/kg 'pre-dose' of either PEGylated- or PTGG-ylated liposomes on day 1 followed by a second dose of 10 μmol of lipid/kg on day 7. After the 10 μmol of lipid/kg (day 1 for 1-dose, or day 7 for 2-dose regime), mice are tail-nicked and ~5 μL of blood was collected in a heparinized capillary tubes at 0.017, 0.25, 0.75, 1.5, 4.5, 24, 48 and 72 hours post-injection. Mice were then sacrificed and their heart, lungs, liver, kidneys and spleen were removed and immediately analyzed for DiD fluorescence using an IVIS system. Using the IVIS Lumina Imaging System (Xenogen Corporation, Alameda, CA, USA) and Living Image software (v4.4), fluorescence was quantified using an excitation wavelength of 660 nm and an emission wavelength of 710 nm. Concurrently, collected blood was diluted into 0.1% Tween 20 and analyzed on a plate-reader spectrofluorimetry (excitation 640 nm, emission 690 nm). Data were fitted to a two-compartment bolus model in PKSolver to determine pharmacokinetic parameters (*n*=4).

Pharmacokinetic study of OVA conjugates in rats. All animal care, housing and experiments were conducted following the guidelines of the Association for Assessment and Accreditation of Laboratory Animal Care (AAALAC), and all procedures were approved by the Rutgers Institutional Animal Care and Use Committee (IACUC).

Sprague Dawley male rats (493±30g) were administered formulations at 500 µg of OVA protein/kg via tail vein injection. Blood samples (200 µL) were collected from the saphenous vein at 0.15, 0.5, 1, 2, 4, 6, 12, 24 and 48 hours after administration. Animals were anesthetized with 1% isoflurane in oxygen during intravenous injection and blood collection. After collecting blood samples, they were centrifuged at 13,200 rpm for 3 min and the plasma was separated and stored at 4ºC until analyzed.

To obtain standard curve for each formulation, 1 µL of each were mixed with 99 µL of plasma and serially diluted and measured by spectrofluorometry (excitation 494 nm, emission 518 nm). Percent injected dose concentration (%ID) at each time point was calculated as: (concentration of each formulation at time x) ÷ (plasma concentration at time 0) x 100. Plasma concentration at t = 0 was determined using the estimations of 64 mL/kg (blood volume) x 0.6 (plasma ratio = 60% of blood) and dividing by injected dose of each formulation (500 µg protein/kg), i.e. $\%ID = \frac{[OVA]_{t=x}}{\frac{OVA\ injected\ (\mu g/kg)}{plasma\ volume}} \times 100$. The pharmacokinetic parameters e.g. half-life (tβ1/2) and Area under the plasma percentage versus time curve (AUC) were calculated using PKSolver as a two-component bolus.[3]

Ethics Statement.

All animal experiments were performed in accordance with National Institutes of Health guidelines for the care and use of laboratory animals. Experimental protocols herein were reviewed and approved by the Institutional Animal and Use Committee (IACUC) at Vanderbilt University (mice) and Rutgers University (rats).

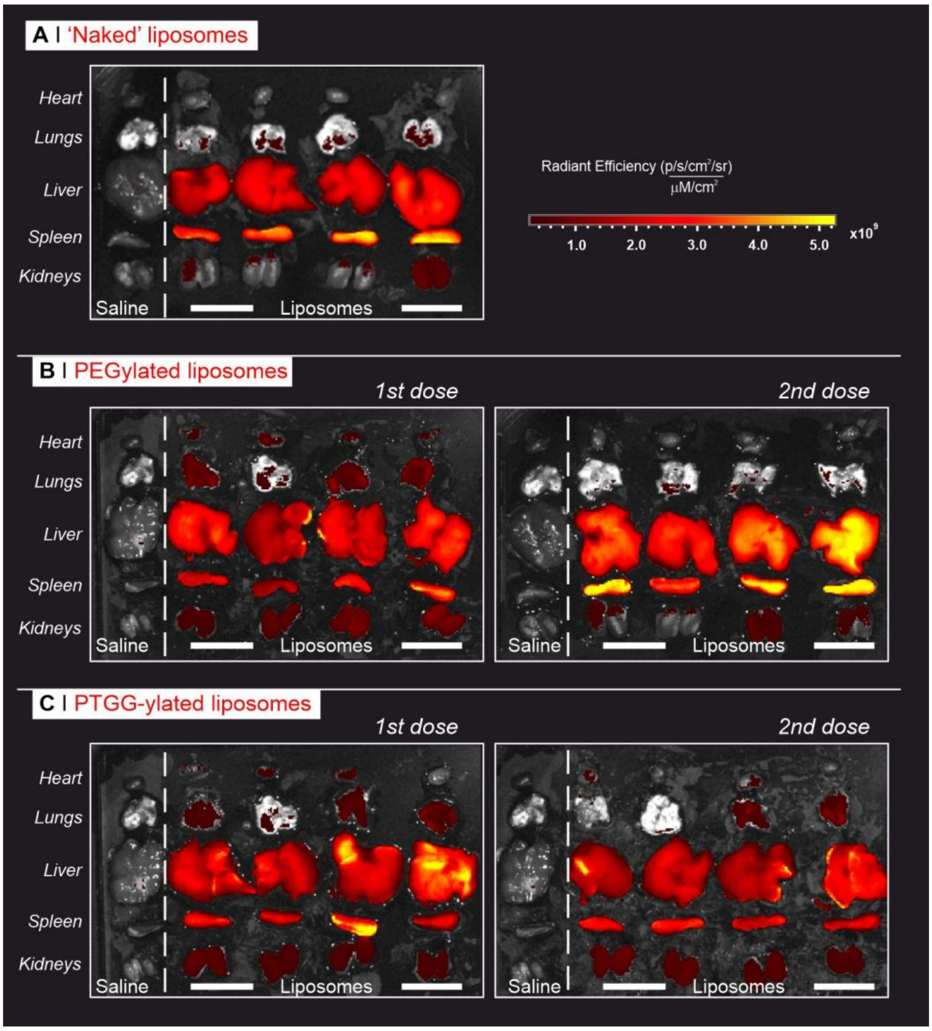

Figure S10. Biodistribution of 1st and 2nd dose liposomes in mice 72 hours after administration. DiD fluorescence in the various organs was imaged using an IVIS system.

References

1. d'Arcy, R.; Tirelli, N., Mitsunobu Reaction: A Versatile Tool for PEG End Functionalization. *Macromol. Rapid Commun* **2015,** *36* (20), 1829-1835.

2. Frey, A.; Di Canzio, J.; Zurakowski, D., A statistically defined endpoint titer determination method for Immunoassays. *J Immunol Methods* **1998,** *221* (1-2), 35-41.

3. Zhang, Y.; Huo, M.; Zhou, J.; Xie, S., PKSolver: An add-in program for pharmacokinetic and pharmacodynamic data analysis in Microsoft Excel. *Computer Methods and Programs in Biomedicine* **2010,** *99* (3), 306-314.
